# Clinical, genetic and pharmacological data support targeting the MEK5/ERK5 module in lung cancer

**DOI:** 10.1038/s41698-021-00218-8

**Published:** 2021-08-17

**Authors:** Adrián Sánchez-Fdez, María Florencia Re-Louhau, Pablo Rodríguez-Núñez, Dolores Ludeña, Sofía Matilla-Almazán, Atanasio Pandiella, Azucena Esparís-Ogando

**Affiliations:** 1 Institute of Molecular and Cellular Biology of Cancer (IBMCC)-CSIC, Salamanca, Spain; 2 Institute of Biomedical Research of Salamanca (IBSAL), Salamanca, Spain; 3 Cancer Network Research (CIBERONC), Salamanca, Spain; 4 Pathology Service, University Hospital, Salamanca, Spain

**Keywords:** Non-small-cell lung cancer, Cell growth, Cell signalling, Targeted therapies

## Abstract

Despite advances in its treatment, lung cancer still represents the most common and lethal tumor. Because of that, efforts to decipher the pathophysiological actors that may promote lung tumor generation/progression are being made, with the final aim of establishing new therapeutic options. Using a transgenic mouse model, we formerly demonstrated that the sole activation of the MEK5/ERK5 MAPK route had a pathophysiological role in the onset of lung adenocarcinomas. Given the prevalence of that disease and its frequent dismal prognosis, our findings opened the possibility of targeting the MEK5/ERK5 route with therapeutic purposes. Here we have explored such possibility. We found that increased levels of MEK5/ERK5 correlated with poor patient prognosis in lung cancer. Moreover, using genetic as well as pharmacological tools, we show that targeting the MEK5/ERK5 route is therapeutically effective in lung cancer. Not only genetic disruption of ERK5 by CRISPR/Cas9 caused a relevant inhibition of tumor growth in vitro and in vivo; such ERK5 deficit augmented the antitumoral effect of agents normally used in the lung cancer clinic. The clinical correlation studies together with the pharmacological and genetic results establish the basis for considering the targeting of the MEK5/ERK5 route in the therapy for lung cancer.

## Introduction

Lung cancer is the most common and lethal tumor^1^. From the clinicopathological point of view, lung tumors are grouped into small cell lung cancer (SCLC) and non-small cell lung cancer (NSCLC)^2^. The former represents around 20% of all lung tumors, is of neural crest origin and responds to chemotherapy. NSCLC accounts for 80% of lung tumors, mainly originates from epithelial cells and the most common subtypes are adenocarcinomas, squamous cell carcinomas, and large cell carcinomas^2^. In spite of advances in treatment strategies with the incorporation of new targeted compounds and immunotherapies, NSCLC has a poor prognosis, particularly in advanced metastatic stages^3^. Therefore, identification of potentially novel actionable targets is required.

Recently, our studies aimed at deciphering the oncogenic properties of the MEK5/ERK5 route, showed that exclusive activation of this pathway provoked lung cancer^4^. The MEK5/ERK5 route belongs to the mitogen-activated protein kinase (MAPK) superclass of signal transduction pathways. It is activated by receptor tyrosine kinases as well as other stimuli, such as changes in osmotic pressure^5^. Stimuli that provoke activation of the route trigger phosphorylation of MEK5 in serine 311 and threonine 315. Such dual phosphorylation causes its activation, and once activated MEK5 phosphorylates ERK5 at threonine 218 and tyrosine 220 (^218^TEY^220^ microdomain), and this increases the activity of ERK5 that then phosphorylates downstream signaling intermediates^6^. In addition, the increase in activity of ERK5 is expected to cause phosphorylation of ERK5 itself at sites located in its C-terminal region, likely through an action of ERK5 in trans^7^.

Activation of the MEK5/ERK5 route has been reported to play a role in the control of cell proliferation^8^. Because of that, various studies have addressed the participation of the MEK5/ERK5 route in cancer, having found that such route is deregulated in several neoplasias [reviewed in refs. ^9–13^]. These studies have been recently complemented with in vivo analyses of the prooncogenic properties of this route^4^. Thus, mice engineered to express a constitutively active form of MEK5 developed lung tumors that were indistinguishable from human lung adenocarcinoma. Biochemically, lung tumors expressing constitutively active MEK5 showed higher levels of constitutive ERK5 activation.

The discovery that activation of the MEK5/ERK5 route acted pathophysiologically in lung cancer in mice, together with the need to develop novel therapeutics for that disease, stimulated a study aimed at exploring the prognostic relevance of MEK5/ERK5 expression in that disorder, as well as the potential therapeutic value of its targeting. We report here that MEK5/ERK5 overexpression is linked to poor patient outcome in lung cancer. Moreover, using pharmacologic tools and genetic approaches we demonstrate that targeting this route may be used therapeutically.

## Results

### MEK5/ERK5 expression linked to patient outcome in lung cancer

**Fig. 1:**
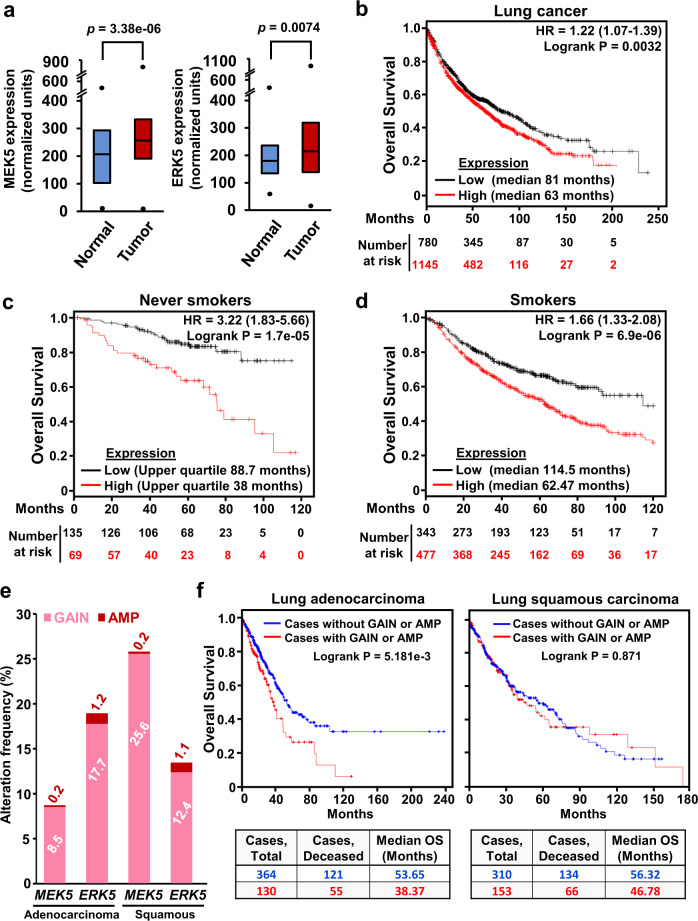
Analysis of the relationship between MEK5/ERK5 expression and overall survival in lung cancer patients. **a** Comparison between MEK5 levels (left panel) of 2437 lung tumors and 86 healthy lung samples plotted from patient data collected in the Kaplan–Meier plotter database. Data were first MAS5 normalized, and then a scaling normalization was performed to set the mean expression across all genes to 1000, as described in ref. ^37^. ERK5 levels (right panel) were similarly represented and compared. Black dots correspond to the minimum and maximum values. *p*-values were calculated according to the Mann–Whitney *U*-test. **b** 240 months follow-up Kaplan–Meier analysis of the relationship between combined MEK5/ERK5 expression and overall survival in lung cancer patients (*n* = 1925) collected in the public Kaplan–Meier plotter database. Patients were stratified according to low or high MEK5/ERK5 expression as indicated in the methods section. The number of patients at risk in the low and high expression groups is shown. **c** 120 months follow up Kaplan–Meier analysis in never-smoking lung cancer patients (*n* = 204) calculated as in (**b**). **d** 120 months follow up Kaplan–Meier analysis in the lung cancer patient population excluding never-smokers (*n* = 820) calculated as in (**b**). **e** The cBioPortal database was explored in order to determine the gains and amplifications present in *MEK5* and *ERK5* genes of lung adenocarcinoma (*n* = 503) and lung squamous cell carcinoma (*n* = 469) patients. Copy-number alteration data was obtained from the TCGA. Patients with gains and high-level amplifications were quantified and represented as percentage from the total patients included in the study. **f** Overall survival curves of lung adenocarcinoma (*n* = 494) and lung squamous carcinoma (*n* = 463) patients with available clinical data from (**e**). Patients harboring gain or amplification in MEK5 or ERK5 (red line) were compared to those without such alterations (blue line). The *p*-value of the studies, follow-up (months) and median overall survival are indicated.

The preclinical precedents linking the MEK5/ERK5 pathway to promotion of lung carcinogenesis in mice^4^ led us to explore its impact on clinical outcome in lung cancer patients. To that end, we first used the Kaplan–Meier plotter online tool that allowed exploration of the mRNA expression levels of lung tumor patient samples (*n* = 2437) compared to normal lung samples (*n* = 86). Quantitative mRNA analyses demonstrated that the levels of MEK5 or ERK5 were higher in tumoral samples than in normal tissue (*p*-value = 3.38e−06 for MEK5 and *p*-value = 0.0074 for ERK5) (Fig. 1a).

After observing that MEK5 or ERK5 expression were higher in tumoral samples than in normal tissue, we next explored the potential relationship between expression levels and patient outcome. mRNA analyses of pooled data from the whole lung cancer patient population present in the database (*n* = 1925) were performed. These studies showed that high levels of MEK5 and ERK5 expression associate with worse prognosis (*p-*value = 0.0032, HR = 1.22 (1.07–1.39), FDR = 0.50) (Fig. 1b), with a median survival of 63 months (high expression cohort), compared to 81 months (low expression cohort). Statistically, this association was more robust when selecting for analysis the adenocarcinoma cohort (*p*-value = 3e−5, HR = 1.65 (1.3–2.1), FDR = 0.01) (Supplementary Fig. 1a). When the squamous carcinoma cohort was selected, such association did not reach statistical significance (*p*-value = 0.07, HR = 0.78 (0.6–1.02), FDR = 1) (Supplementary Fig. 1b).

The negative association between high levels of MEK5/ERK5 expression and worse prognosis was observed irrespectively of whether lung cancer patients did not have previous history of tobacco smoking (*n* = 204, *p-*value = 1.7e−05, HR = 3.22 (1.83–5.66), FDR = 0.01) (Fig. 1c), or were smokers (*n* = 820, *p-*value = 6.9e−06, HR = 1.66 (1.33–2.08), FDR = 0.01) (Fig. 1d). Complementary studies indicated that MEK5 and ERK5 high expression was also associated with a poor post progression survival prognosis (Supplementary Fig. 1c) whose median survival was reduced by more than half in the never-smoking cohort of patients (*p-*value = 0.029, HR = 1.98 (1.06–3.7), FDR = 0.50).

The potential correlation between MEK5 and ERK5 expression and patient outcome was also explored using the cBioPortal for Cancer Genomics database which, in addition to mRNA expression levels, allows analysis of gene copy number alterations. Gain or amplification of *MEK5* were present in 8.7% of adenocarcinomas and 25.8% of squamous carcinomas, while in the case of *ERK5* such molecular alterations were present in 18.9% of adenocarcinomas and 13.5% of squamous carcinomas (Fig. 1e). Lung adenocarcinoma patients presenting amplifications or gains in *MEK5* or *ERK5* showed a worse clinical outcome when compared to those without such alterations (*p*-value = 5.181e−3) (Fig. 1f). However, no significant differences were observed in the clinical outcome of lung squamous carcinoma patients. As expected, the cases with gains or amplifications presented the highest mRNA levels in lung adenocarcinomas as well as squamous cell tumors (Supplementary Fig. 2a), that are the main lung cancer subtypes. Similar analyses of *MEK5* or *ERK5* gene deletions failed to show association between these molecular alterations and patient outcome (Supplementary Figs. 2b–d). Together, these clinic-genomic studies linked high expression levels of MEK5 or ERK5 to worse patient outcome in lung cancer, especially in lung adenocarcinoma patients.

### The MEK5/ERK5 axis regulates proliferation of lung cancer cells

The link between MEK5/ERK5 expression levels and patient outcome led us to explore the value of targeting such route as a potential therapeutic strategy using non-small cell lung cancer (NSCLC) cell lines. To that end, we aimed at selecting in vitro cellular models that in addition to expressing ERK5, would also show evidence of MEK5/ERK5 pathway activation. Of a panel of 14 NSCLC cell lines, all expressing ERK5, we selected the four ones (A549, H1299, H460, and H727) that constitutively expressed pERK5 (Supplementary Fig. 3a). The latter represents a readout of MEK5/ERK5 pathway activation status^8,14–16^.

**Fig. 2:**
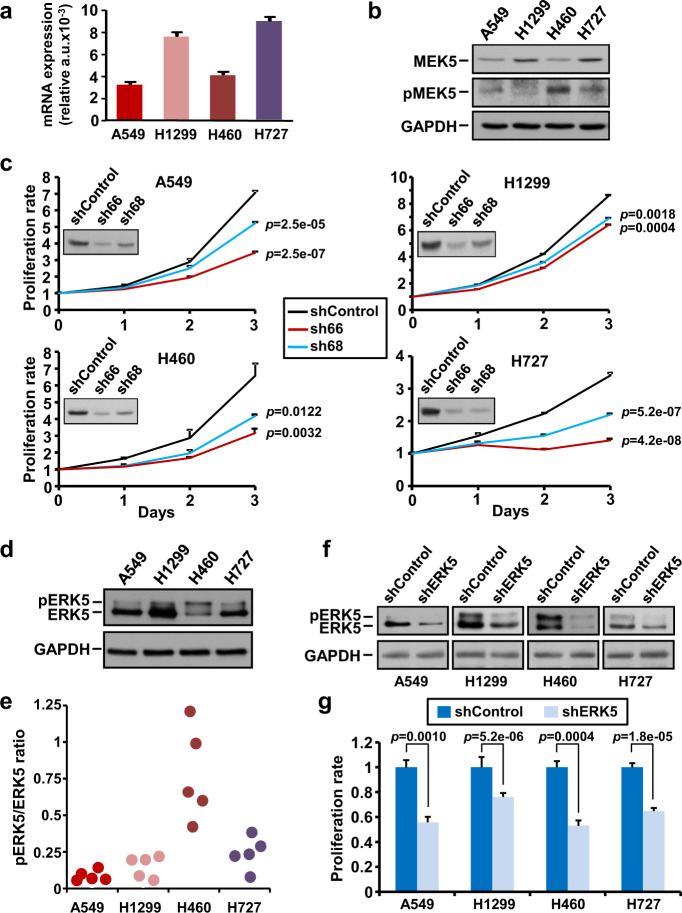
Effect of the MEK5/ERK5 knockdown on cell proliferation of lung cancer cells. **a** MEK5 mRNA expression was determined by quantitative RT-PCR in NSCLC cell lines. GAPDH was used as control for differences in cDNA input. Data show the mean expression ± SD of an experiment that was repeated twice, calculated according to the ΔΔC_t_ relative quantitation method. **b** Expression of MEK5 and pMEK5 in NSCLC cell lines. 50 µg of whole-cell lysates were used to detect MEK5 by western blotting. For pMEK5 detection, 2 mg of protein were immunoprecipitated with the anti-MEK5 antibody followed by western blotting with the anti-pMEK5 antibody. GAPDH was used as loading control. **c** Effect of MEK5 knockdown on cell proliferation. Knockdown of MEK5 was carried out by lentiviral infection of NSCLC cells with a scrambled control sequence or MEK5 specific shRNAs (sh66 and sh68) and levels of MEK5 were evaluated by western blotting. The effect of MEK5 knockdown on cell proliferation was measured by MTT assay at the indicated times. Data are presented as the mean ± SD of an experiment that was repeated three times. *p*-values were calculated according to a two-sided Student’s *t*-test. **d** Expression of total ERK5 in NSCLC cell lines. pERK5 and total ERK5 were evaluated by immunoprecipitating 1 mg of protein with the anti-ERK5 antibody and blots probed with the C-terminal anti-ERK5 antibody. GAPDH was used as loading control. **e** pERK5 quantitation in NSCLC cells. pERK5 (upper band) and ERK5 (lower band) levels from five independent western blot studies were quantified with the ImageJ software and the pERK5/ERK5 ratio represented. **f** Silencing of ERK5 in NSCLC cells by shRNA was confirmed by western blotting with the anti-ERK5 antibody. GAPDH was used as loading control. **g** Proliferation effect of ERK5 knockdown was evaluated at 3 days as in (**c**).

Quantitative PCR (Fig. 2a) and western blot analyses of MEK5 (Fig. 2b) demonstrated expression of this protein in A549, H1299, H460, and H727 NSCLC cell lines. Using an antibody raised to residues pSer^311^ and pThr^315^ present in the MEK5 activation domain, dually phosphorylated MEK5 was detected in these cell lines, although at different levels (Fig. 2b). Lentiviral vectors including sequences expected to interfere with MEK5 expression were used with the purpose of establishing the potential role of that kinase in lung cancer cell proliferation. The four sequences explored were able to reduce MEK5 expression (Supplementary Fig. 3b). However, the largest interference was observed in cells infected with vectors including sequences sh66 and sh68. Knockdown of MEK5, resulted in significant reduction of the proliferation of the four lung cancer cell lines (Fig. 2c). Of note, knockdown of MEK5 caused a reduction of pERK5 levels (Supplementary Fig. 3c) which correlated with inhibition of proliferation (Fig. 2c) in all four NCSLC cells.

The relevance of ERK5 in lung cancer cell proliferation was also explored. Western blotting studies revealed that all cell lines studied expressed pERK5, with H460 cells showing the highest pERK5/ERK5 ratio (Fig. 2d and e). Knockdown of ERK5 reduced the amounts of both ERK5 and pERK5 (Fig. 2f) and significantly decreased the proliferation rates of the four cell lines (Fig. 2g). The participation of the MEK5/ERK5 route in the proliferation of non-transformed immortalized human bronchial epithelial cells (HBEC) was also evaluated. MEK5 or ERK5 knockdown decreased proliferation of HBEC (Supplementary Fig. 3d and e), suggesting an important role of this pathway in the control of the proliferation of non-transformed lung epithelial cells.

**Fig. 3:**
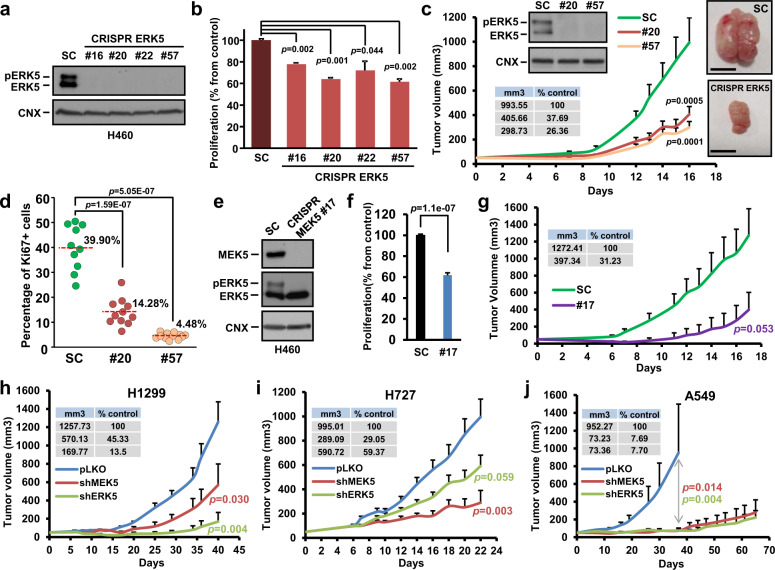
Effect of ERK5 knockout on tumor growth of H460 lung cancer cells. **a** Western blot showing lack of ERK5 expression in the different H460 ERK5 CRISPR clones when compared to the control H460 scramble cells (SC). **b** 35,000 cells of H460 SC or H460 CRISPR ERK5 clones were cultured for 3 days in p6 wells and the proliferation rate was measured by cell counting. Data are represented as mean percentage from control H460 scramble cells ± SD of an experiment that was repeated three times. *p*-values are indicated. **c** Tumor growth evolution of the mice xenoinjected with H460 SC or ERK5 CRISPR cells (#20 and #57 clones) (*n* = 10 tumors, per group). The mean tumor volume and SEM error bars of each group are represented. The experiment was stopped once one of the mice tumors reached a volume of 1500 mm^3^. The volume percentage of ERK5 CRISPR tumors when compared to scramble tumors, the significative *p*-values, and representative pictures are displayed (scale bars = 1 cm). Inset western blot showing that ERK5 expression was not recovered in the H460 ERK5 CRISPR cells after the in vivo experiment is included. **d** Ki67 immunostaining was quantified from 10 to 12 different pictures of scramble and ERK5 CRISPR clones and represented as percentage of Ki67 positive cells from total. The average percentage (red line) and *p*-value for each ERK5 CRISPR clone are indicated. **e** Western blot analyses of MEK5, ERK5, and pERK5 in the H460 MEK5 knockout clone #17. **f** In vitro and **g** in vivo effect of the MEK5 knockout on the proliferation and tumorigenesis of H460 cells (*n* = 7 tumors per group). The procedures for evaluation of these effects were as described above for ERK5. **h** Effect of MEK5 or ERK5 knockdown on the in vivo tumorigenesis of H1299 cells (*n* = 5–6 tumors per group), **i** H727 cells and **j** A549 cells (*n* = 6–8 tumors per group). Equal number of pLKO shControl, shMEK5 (sh66) or shERK5 cells were injected subcutaneously, and tumor sizes periodically measured. The mean tumor volume, SEM error bars, and *p*-values between groups are indicated. Additional information is provided in the “Methods” section.

We also used the CRISPR/Cas9 technique to complement the knockdown studies on the relevance of MEK5 and ERK5 in lung cancer cell proliferation. For these studies we selected the H460 cell line, which presented the highest ratio of ERK5 activation (Fig. 2e). Four clones of H460 CRISPR cells lacked ERK5 by western blotting (Fig. 3a). In vitro cell proliferation studies demonstrated that elimination of ERK5 expression significantly affected the proliferation of H460 cells (Fig. 3b). Transcriptomic analyses showed that the ERK5 knockout clones were different from each other (data not shown), excluding that their proliferation characteristics were due to a clonal effect.

The antiproliferative action of ERK5 deletion was also explored in a more physiological context. To that end, H460 ERK5-KO cells from two different clones (#20 and #57) were injected into mice, and the growth of tumors generated by those cells compared to the growth of tumors created by injecting H460 control cells. As shown in Fig. 3c, tumors in mice injected with ERK5 knockout cells grew slower than those created by injecting H460 control cells. Western blot analyses of the tumors dissected from these animals demonstrated the lack of expression of ERK5 in the knockout clones (inset in Fig. 3c). Moreover, Ki67 quantitative immunostaining of tumoral samples indicated that the proliferation rate was significantly lower in H460 ERK5-KO than in control H460 tumors (Fig. 3d). As a complement to these studies, we analyzed the proliferation and tumorigenic properties of a MEK5 knockout clone derived from H460 cells (Fig. 3e). Elimination of MEK5 resulted in disappearance of pERK5. The MEK5 knocked out clone (#17) grew less than the SC H460 isogenic control cells, which expressed MEK5 (Fig. 3f). Moreover, in vivo experiments showed that tumors in mice caused by the injection of cells from the MEK5 knockout clone grew substantially less than tumors created by injection of control SC cells (Fig. 3g).

Finally, we tested the effect of knocking down MEK5 or ERK5 on the growth of tumors derived from injection of H1299, H727, and A549. As shown in Fig. 3h–j, injection of cells in which MEK5 or ERK5 were knocked down by using lentiviral shRNA resulted in a much slower growth of the tumors, as compared to tumors created by cells infected with a lentiviral control plasmid (pLKO shControl).

### Pharmacological inhibition of MEK5 or ERK5 in lung cancer cells

**Fig. 4:**
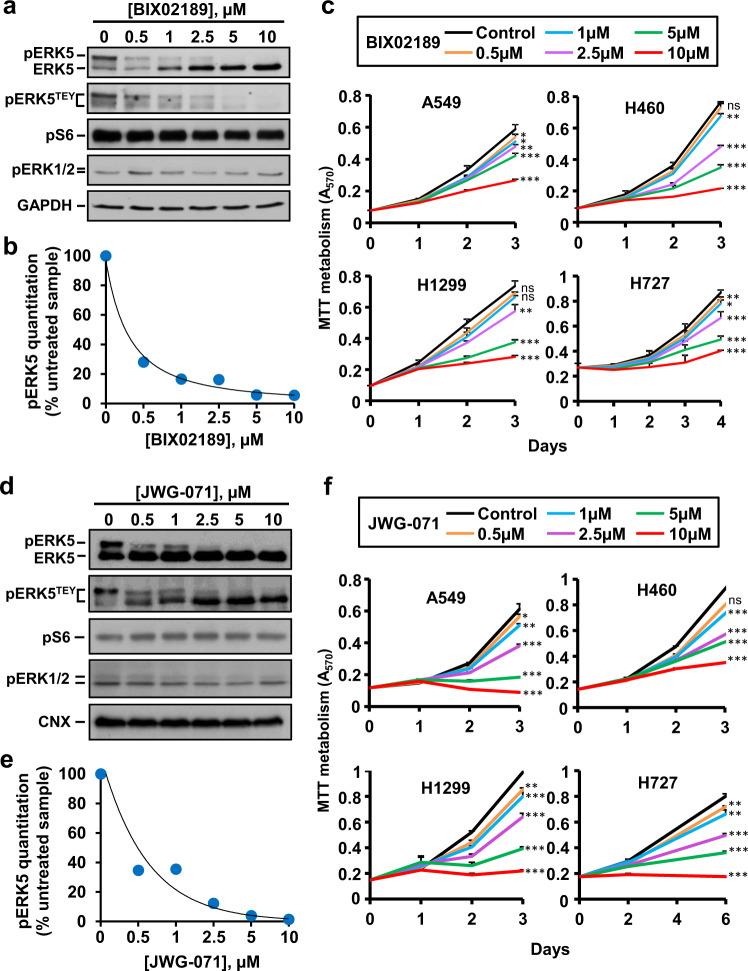
MEK5 and ERK5 inhibitors reduced cell proliferation of lung cancer cells. **a** H460 cells were treated with increasing doses of BIX02189 for 72 h and the effect of the drug on ERK5 activation was evaluated by immunoprecipitation of 1 mg of whole-cell lysates and western blotting with the anti-ERK5 or anti-pERK5^TEY^ antibodies. pS6 and pERK1/2 were used as controls to assure that BIX02189 did not affect the PI3K and ERK1/2 routes. GAPDH was used as loading control. **b** Quantitation of pERK5 band in H460 cells from (**a**) using the ImageJ software. Data represent the percentage of the upper pERK5 band after BIX02189 treatment with respect to such band in control H460 untreated cells. **c** NSCLC cells were plated in 24-well dishes and treated with increasing doses of BIX02189. Cell proliferation was measured at the indicated times by MTT. Results are expressed as mean ± SD of an experiment that was repeated three times. *p ≤ 0.05; **p ≤ 0.01; ***p ≤ 0.001. The exact *p*-values are shown in Supplementary Table 1a. **d** H460 cells were treated with increasing doses of JWG-071 for 7 h. Inhibition of ERK5 activation was evaluated by immunoprecipitation of 1.3 mg of whole-cell lysates with the anti-ERK5 antibody and probed with the C terminal anti-ERK5 or anti-pERK5^TEY^ antibodies. pS6 and pERK1/2 were used as controls to assure that JWG-071 did not affect the PI3K and ERK1/2 routes. GAPDH was used as loading control. **e** Quantitation of pERK5 bands was performed as in (**b**). **f** NSCLC cells were plated in 24-well dishes and treated with increasing doses of JWG-071. Cell proliferation was measured at the indicated times by MTT. Results are expressed as mean ± SD of an experiment that was repeated three times. *p ≤ 0.05; **p ≤ 0.01; ***p ≤ 0.001. The exact *p*-values are shown in Supplementary Table 1b.

With the aim of exploring the effect of MEK5 pharmacological targeting in lung cancer, we evaluated the action of BIX02189, a small molecule kinase inhibitor of MEK5^17^. First, we analyzed the biochemical effect of that inhibitor on ERK5 phosphorylation (as a readout of MEK5 activity) by using the H460 cell line (Fig. 2d and e). Western blotting with antibodies that recognize total or ERK5 phosphorylated at its activation microdomain showed that BIX02189 caused a dose-dependent inhibition of ERK5 phosphorylation, demonstrating that the agent effectively neutralized the upstream kinase MEK5 (Fig. 4a and b). In contrast, the drug did not affect the phosphorylation levels of pERK1/2 or pS6, used as surrogate markers for activation of the RAS-RAF and PI3K/AKT routes, respectively, supporting that the drug did not affect the kinase pathways controlling phosphorylation of these signaling mediators. BIX02189 was able to impede ERK5 activation in the four cell lines (Supplementary Fig. 4a and b). The drug caused a dose-dependent (Fig. 4c) and significant (Supplementary Table 1a) decrease in MTT metabolization, indicating the relevance of the activity of this kinase in lung cancer cell proliferation.

The value of targeting the kinase activity of ERK5 using the inhibitor JWG-071^18^ was also explored. Western blotting analysis with the anti-ERK5 or anti-pERK5^TEY^ antibodies showed that treatment of H460 cells with JWG-071 dose-dependently decreased the mobility of ERK5 towards a faster migrating form, indicating that the drug inhibited hyperphosphorylation of ERK5 (Fig. 4d and e and Supplementary Fig. 4c and d). Of note, such inhibition of phosphorylation did not appear to affect the phosphorylation of the TEY microdomain, since the anti-pERK5^TEY^ antibody was still able to detect the faster migrating ERK5 forms. These results demonstrate that the drug was able to in vivo neutralize ERK5 autophosphorylation but did not affect the phosphorylation of ERK5 at the TEY microdomain, which is due to the activity of MEK5. Moreover, the data also demonstrate that the gel shift of ERK5 towards a lower mobility is not solely caused by phosphorylation of ERK5 at the TEY microdomain, since the faster migrating ERK5 is still recognized by the antibody. JWG-071 did not affect pS6 or pERK1/2 phosphorylations. Biologically, JWG-071 decreased, in a dose and time-dependent fashion, the proliferation of the four lung cancer cell lines (Fig. 4f and Supplementary Table 1b). Similar biochemical (Supplementary Fig. 4e and f) and biological (Supplementary Fig. 4g and Supplementary Table 1c) results were obtained by using XMD8-92, an alternative ERK5 inhibitor^19^.

### MEK5 inhibition slows cell cycle progression

**Fig. 5:**
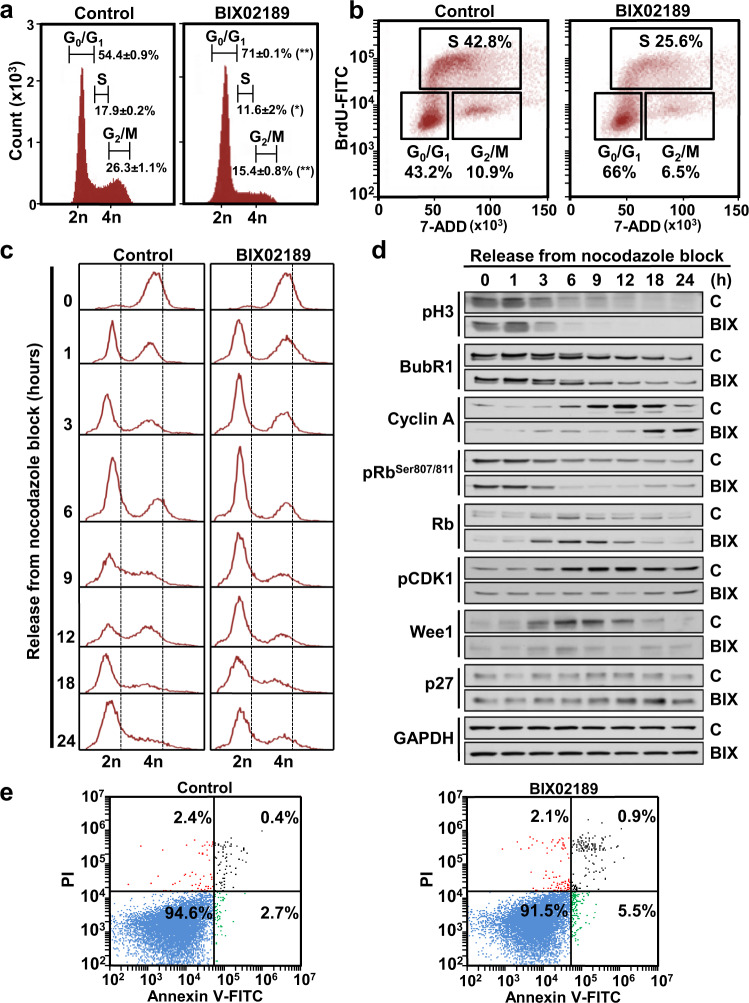
Influence of MEK5 activity-inhibition on cell cycle progression. **a** Cell cycle profile of H460 cells treated with 5 µM BIX02189 and analyzed by FACS at 24 h. Results are expressed as mean percentage of cells in each cell cycle phase ± SD of an experiment that was repeated three times. *p ≤ 0.05; **p ≤ 0.01 for the statistical comparison between the same cell cycle phases of control or BIX02189-treated samples. Exact *p*-values are shown in Supplementary Table 1d. **b** BrdU incorporation was coupled to 7-ADD staining and measured at 24 h using the FITC BrdU Flow Kit to precisely define the percentage of cells undergoing DNA synthesis. **c** H460 cells were synchronized in mitosis by nocodazole. Mitotic cells were released from nocodazole block in the absence or presence of 5 µM BIX02189 for the indicated times and the effect of the drug on the different phases of cell cycle was analyzed by FACS, and **d** proteins implicated in cell cycle progression were evaluated by western blotting with the indicated antibodies. GAPDH was used as loading control. **e** H460 cells were either untreated or treated with 5 μM BIX02189 for 72 h and apoptosis was analyzed by Annexin V-FITC assay.

The decrease in MTT metabolism values caused by BIX02189 could be due to decreased proliferation, increased cell death, or both. Propidium iodide staining showed that treatment with BIX02189 significantly increased the number of cells present in the G1 phase of the cell cycle (Fig. 5a and Supplementary Table 1d). The drug decreased DNA synthesis of asynchronous H460 cells, as demonstrated by pulse labeling with BrdU followed by cytometric analysis (Fig. 5b). The delay in cell cycle progression was confirmed in cell cycle synchronization experiments. H460 cells were synchronized in mitosis by incubation with nocodazole, and then released in the absence or presence of BIX02189. Parallel cultures of cells were analyzed cytometrically (Fig. 5c) and by western blotting (Fig. 5d). H460 cells were arrested in M upon incubation with nocodazole as demonstrated by accumulation of phosphorylated histone H3 as well as phosphorylated BubR1, two proteins whose phosphorylation is used as readout for cells in mitosis (Fig. 5d). One hour after release from the nocodazole block, a substantial amount of cells had progressed into G1, and such progression was unaffected by BIX02189 (Fig. 5c), suggesting that exit from mitosis is unaffected by the drug. The histograms obtained at 9 and 12 h after release showed that BIX02189-treated cultures accumulated in the G1 phase and did not efficiently progress along the S phase when compared to untreated cells. Biochemically, BIX02189 inhibited phosphorylation of Rb at serines 807/811 (Fig. 5d and Supplementary Fig. 5a). Such phosphorylations are required for adequate progression through G1^20^. As a result of such inhibition, the levels of total underphosphorylated Rb were higher in cells treated with the drug. p27 levels were also increased in cells incubated with BIX02189. In addition, cyclin A levels increased at earlier times in control cells than in BIX02189-treated cells (Fig. 5d and Supplementary Fig. 5a). The levels of Wee1 and pCDK1, two proteins used as markers of G2/M transition^21^, were also downregulated by treatment with the drug, reflecting the defective progression of cells along the cell cycle, which decreased the reaching of H460-treated cells to the G2 phase of the cell cycle.

To explore whether increased cell death contributed to the mechanism of the antiproliferative action of BIX02189, H460 cells were treated with the drug and Annexin V/propidium iodide staining performed. Treatment for up to three days with the drug did not increase Annexin V-positive staining (Fig. 5e). Biochemical analyses of indicators of apoptotic cell death also failed to show an appreciable effect of the drug on their levels (Supplementary Fig. 5b) or caspase 8 and caspase 3 activities (Supplementary Fig. 5c). These data suggested that stimulation of cell death did not participate in the antitumoral action of the drug.

### Effect of MEK5/ERK5 targeting on the antitumoral action of standard of care drugs

**Fig. 6:**
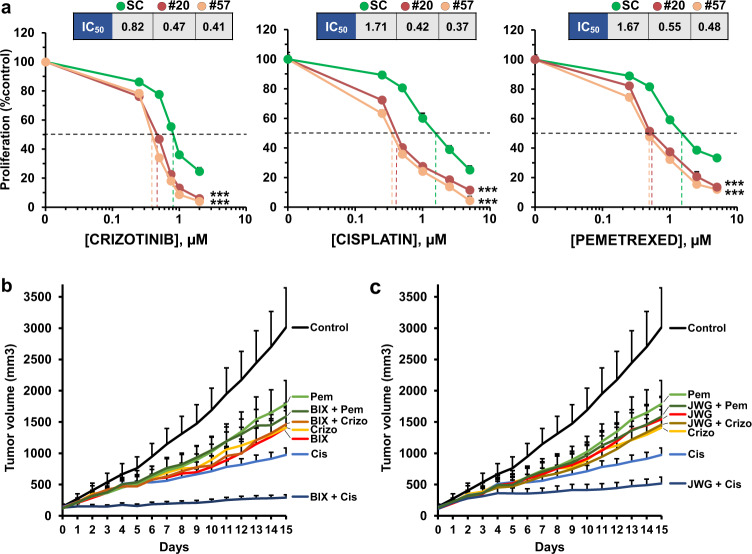
MEK5/ERK5 inhibition synergizes with standard of care treatments. **a** H460 scramble cells and H460 ERK5 CRISPR clones (#20 and #57) were plated in 24-well dishes, and 24 h later treated with the indicated doses of standard of care agents for 48 h. Cell viability was measured by an MTT assay and each condition (scramble, #20 and #57) was represented as percentage from their respective untreated cells. Results are expressed as mean ± SD of an experiment that was repeated twice. Error bars are shown. The IC50 values and *p*-values for each drug and condition are also indicated (***p ≤ 0.001). **b** In vivo analyses of the effect of BIX02189 and JWG-071 (**c**) alone or in combination with standard of care drugs, on the growth of H460 cells subcutaneously implanted in mice and treated with the indicated drugs as described under the “Methods” section. Tumor masses (*n* = 8–10 tumors per group) were measured daily for two weeks. The mean tumor volume and SEM error bars of each group are represented. To avoid excessive overlapping of the curves, the data of the drug combination with BIX02189 or JWG-071 were split in (**b**, **c**). Therefore, please notice that the curves for control, pemetrexed, crizotinib or cisplatin are the same in both (**b**, **c**). Exact *p*-values of comparisons between control untreated and treated groups are shown in Supplementary Table 1e and g.

Since most therapeutic regimens in oncology include combinations of drugs to increase antitumoral responses, we next explored whether neutralization of the MEK5/ERK5 route could increase the action of standard of care drugs used in the lung cancer clinic. To this end, the impact of ERK5 loss of function on drug antitumoral efficacy was analyzed comparing the response of H460 ERK5-KO cells to the response of H460 cells expressing the kinase. H460 SC or H460 ERK5-KO cells were treated with different doses of chemotherapeutic agents (cisplatin and pemetrexed) as well as targeted therapies (crizotinib, since H460 cells express active ALK) and their proliferation measured. Deletion of ERK5 led to an increase in the efficacy of all treatments, especially in the case of cisplatin (Fig. 6a). The IC_50_ values were reduced to the half or even more in the H460 ERK5-KO clones when compared to the H460 control cells.

The effect of MEK5 or ERK5 inhibitors either alone or in combination with standard of care drugs was evaluated in mice injected with H460 cells. Treatment with BIX02189 or JWG-071 decreased the pERK5/ERK5 ratio, indicating that these drugs were also able to inhibit MEK5/ERK5 pathway activation in vivo (Supplementary Fig. 6a and b). BIX02189 and JWG-071 provoked a significant reduction in tumor burden (Fig. 6b and c and Supplementary Table 1e). Of note, these drugs exerted a tumor growth inhibitory effect similar to the standard of care drugs (Fig. 6b and c and Supplementary Table 1f). These in vivo experiments showed that combination of BIX02189 or JWG-071 with cisplatin exerted a significant antitumoral effect on the growth of the H460 xenografts, which was also significantly higher than the effect of the individual treatments (Supplementary Table 1g).

## Discussion

Despite therapeutic advances achieved in the last years, lung cancer leads the list of most lethal tumors^1,2^. Therefore, there is still a great need to uncover the pathophysiological bases of lung tumors to guide in the development of new therapies^22^. Based on the precedent that transgenic mice expressing a constitutively active MEK5 form developed lung adenocarcinomas, we decided to explore the potential relationship between expression of the pathway components MEK5 and ERK5 and patient outcome. Moreover, using genetic and pharmacological tools we have evaluated the value of targeting this route with therapeutic purposes.

In silico analyses of public databases pointed out that increased levels of MEK5/ERK5 are associated with worse outcome. It is of interest to note that this association reduced approximately by half the survival of the high MEK5/ERK5 expression subsets of lung cancer patients, irrespective of their smoking history. Several circumstances may explain the activation/overexpression of MEK5/ERK5 pathway in lung cancer. Fluorescence in situ hybridization analyses identified *ERK5* gene amplification in 4% of non-small cell lung cancers^23^. Gene amplification provokes increased expression that in turn may favor pathway activation. In fact, overexpression of ERK5 has also been reported in triple-negative breast tumors and is associated with increased activity of the pathway^15^. It is important to indicate that previous studies have shown that high levels of MEK5 are accompanied by pathway activation in lung adenocarcinoma^4^, pointing to a link between high expression levels of MEK5/ERK5 and pathway functionality. An amplification of the region where *ERK5* is located has been detected in approximately 50% of primary hepatocellular adenocarcinoma tumors^24^ and recently it has been shown that ERK5 modulates tumor development in this type of carcinoma^16^. Other genetic studies have linked the MEK5/ERK5 route to lung cancer. Thus, genome-wide association studies (GWAS) revealed that the chromosomal regions in which the *MEK5* (15q23) and *ERK5* (17p11.2) genes are located are potentially susceptible regions of lung cancer risk^25^. In addition, a recent study demonstrated that an atypical allele polymorphism in the *ERK5* gene in the Chinese population was associated with increased lung cancer risk in smokers^26^. It is noteworthy to comment that exposure to cigarette smoke, a common risk factor for lung cancer, leads to ERK5 activation^27^. Moreover, certain EGFR mutants such as the G719S and L858R found in lung cancer have been reported to activate the MEK5/ERK5 route^28^. While our study did not focus on the analysis of a potential link between oncogene-driven lung cancer and the MEK5/ERK5 route, the fact that RAS^29^, EGFR^8^, or ALK^30^ have been reported to activate that pathway raises the interesting possibility that prooncogenic signaling by those drivers may use the MEK5/ERK5 route. That hypothesis deserves careful study, especially considering potential therapeutic implications. It is also relevant to mention that analyses of the TCGA PanCancer Atlas study (*n* = 1144) revealed mutation frequencies of 0.6% and 0.9% in *MEK5* and *ERK5*, respectively, in lung cancer patients. The relevance of those mutations on patient outcome or their impact on the function of these kinases remains to be established.

In vitro and in vivo studies using different NSCLC cell lines demonstrated the relevance of this pathway in lung cancer proliferation and tumorigenesis. Knockdown experiments revealed that MEK5 or ERK5 were required for the proliferation of lung cancer cells in vitro and for their tumorigenic potential in vivo. In addition, knockout of ERK5 or MEK5 also resulted in decreased cell proliferation in vitro and in vivo. In the latter models, the lack of ERK5 or MEK5 profoundly influenced tumor growth and had a significant effect on the proliferation marker Ki67. Of note, in those in vivo models, the antitumoral effect of ERK5 or MEK5 deletions was severe, underlining the relevance of the MEK5/ERK5 route in the pathophysiology of these tumors. In fact, ERK5 has been reported to facilitate in vivo angiogenesis^31^, which is expected to contribute to the increase in size of tumors.

In line with the genetic data were the findings obtained with drugs that target MEK5 or ERK5. Thus, in vitro pharmacologic studies carried out with MEK5 or ERK5 inhibitors indicated that these drugs inhibited pathway activity and also inhibited the proliferation of lung cancer cell lines. Moreover, in vitro genetic-pharmacologic studies showed that deletion of *ERK5* augmented the antitumoral effect of therapy currently used in the lung cancer clinic. Such effect was especially relevant in the case of cisplatin, whose antitumoral action was also potentiated in vivo by pharmacological targeting of MEK5 or ERK5. The latter is particularly important, since increases in the action of standard of care drugs is desirable for the clinical development of novel drugs.

Mechanistic studies indicated that the MEK5 inhibitor exerted its antiproliferative effect by slowing progression of cells along the G1 phase into S. Such delay in progression was confirmed in cell cycle synchronization experiments. In cells treated with BIX02189, the increase in cyclin A was substantially delayed with respect to control untreated cells. In addition, phosphorylation of Rb at serines 807/811, which is controlled by CDKs 2, 3, 4, and 6 and is required for adequate cell cycle progression^32–34^, was inhibited by the drug. These data indicate that progression of lung cancer cells along the cell cycle requires integrity of the MEK5/ERK5 pathway.

In conclusion, the clinical and the functional data we show here support that targeting the MEK5/ERK5 route may represent a novel therapeutically relevant strategy worth being explored in lung cancer, particularly in combination with standard of care drugs. Such strategy requires identification of patients which may benefit from therapies that target this pathway. In this regard, the studies presented here show a relation between high levels of MEK5/ERK5 and patient outcome. Therefore, analyses of those levels could be used as an initial step to select patients for a potential therapy targeting this pathway. This criterion could be complemented with an analysis of MEK5/ERK5 pathway activation. At present, the best readout of pathway activation is the analysis of pERK5. However, measurement of pERK5 in patient samples is limited by two factors. First, routine studies of pERK5 by western blotting in patient samples appears more complicated than immunohistochemical analyses, as it requires fresh samples and a procedure not well standardized across pathology laboratories. On the other hand, one of the limitations of the use of anti-pERK5 antibodies in immunohistochemistry is their cross-reactivity with pERK1/2. A possible alternative to these methods could be the identification of genes or genesets whose expression would indicate a pathophysiological role of MEK5/ERK5 in tumor progression. Studies to identify an adequate way of biomarking this route with the purpose of patient selection are now being carried out in our laboratory. Besides definition of patients that may benefit from targeting the MEK5/ERK5 pathway, efforts directed at developing clinical stage drugs that specifically target this pathway should offer novel therapeutic opportunities to fight lung cancer and potentially other tumors in which this pathway plays a pathophysiological role.

## Methods

### Reagents and immunochemicals

BIX02189 and crizotinib were purchased from Selleckchem (Houston, TX, USA), XMD8-92 was from Tocris (Bristol, UK), cisplatin from Pharmacia (Peapack, NJ, USA), pemetrexed from Eli Lilly (Indianapolis, IN, USA), and JWG-071 was initially provided by Dr. Nathanael S. Gray (Dana-Farber Cancer Institute, Boston, MA, USA) and later purchased from Glixx Laboratories (Hopkinton, MA, USA). The antibodies utilized along this work including their manufacturer, catalog number and dilution are as follows:

MEK5: MEK5 polyclonal antibody; cat. n° ADI-KAP-MA003; lot. n° 01031958 (dilution 1:3000). Enzo life Sciences (Farmingdale, NY. USA).

GAPDH: GAPDH (FL-335) mouse polyclonal; cat. n° sc-25778; lot n° J0212 (dilution 1:20,000). Santa Cruz Biotechnology (Santa Cruz, CA, USA).

PARP: PARP-1 (F-2); cat. n° sc-8007; lot n° A0617 (dilution 1:2500). Santa Cruz Biotechnology.

pCDK1: p-Cdc2 p34 (Tyr 15); sc-7989 (dilution 1:6000). Santa Cruz Biotechnology.

Wee1: Wee1 (H-300); cat. n° sc-9037; lot n° L1905 (dilution 1:1000). Santa Cruz Biotechnology.

Cleaved Caspase-3: Cleaved Caspase-3 (Asp 175) (5A1E) rabbit mAb; cat. n° 9664; lot n° 21 (dilution 1:2000). Cell Signaling Technology (Danvers, MA, USA).

pERK1/2: Phospho-p44/42 MAPK (ERK1/2) (Thr202/Tyr204) (E10) Mouse mAb; cat. n° 9106; lot n° 43 (dilution 1:3000). Cell Signaling Technology.

pS6: Phospho-S6 Ribosomal Protein (Ser240/244) Antibody; cat. n° 2215 (dilution 1:10000). Cell Signaling Technology.

p27: p27 Kip1 (D69C12) XP® Rabbit mAb; cat. n° 3686; lot n° 5 (dilution 1:3000). Cell Signaling Technology.

Caspase-8: Caspase-8; cat. n° 551242 (dilution 1:1000); BD Biosciences (San Jose, CA, USA).

Caspase-3: Purified Mouse Anti-Human Caspase-3, Monoclonal (19/Caspase-3/CPP32); cat n° 610323; lot n° 17922 (dilution 1:5000). BD Biosciences.

Cyclin A: Purified Mouse Anti-Human Cyclin A, Clone 25/Cyclin A; cat n° 611268; lot n° 5023928 (dilution 1:5000). BD Biosciences.

BUBR1: Purified Mouse Anti-Human BUBR1, Clone 9/BUBR1; cat n° 612503 (dilution 1:3500); BD Biosciences.

Rb: Purified Mouse Anti-Human Retinoblastoma Protein, Monoclonal (G3-245); cat. n° 554136. Lot n° 36721 (dilution 1:2000). BD Biosciences.

pRb: Purified Mouse anti-Rb (pS807/pS811); cat n° 558389 (dilution 1:2000); BD Biosciences.

Calnexin: Rabbit polyclonal anti-calnexin; cat. n° SPA-860; lot n° B509422 (dilution 1:40000). Stressgen Bioreagents (Victoria, BC, Canada).

Ki-67: Rabbit Anti-Human Ki-67 Monoclonal Antibody (Clone SP6); cat. n° MAD-000310QD. Vitro Master Diagnostica (Granada, Spain).

pH3: Anti-phospho-Histone H3 (Ser10) Antibody, Mitosis Marker; cat. n° 06-570; lot n° 3076467 (dilution 1:2500). EMD Millipore Corp. (Billerica, MA, USA).

Antibodies recognizing ERK5 or pERK5 have been previously described^14,35^. The anti-pMEK5 antibody was developed in our laboratory in rabbits against an epitope containing pSer^311^ and pThr^315^. Horseradish peroxidase-conjugated secondary antibodies were from Bio-Rad (Hercules, CA, USA).

Cell culture reagents and other generic chemicals were purchased from Invitrogen (Carlsbad, CA, USA). Sigma-Aldrich (St Louis, MO, USA), BD Biosciences (San Jose, CA, USA) or Merck (Darmstadt, Germany).

### Cell culture and lentiviral infection

A549, H1299, H460, H727, H441, H820, H1404, H23, H1975, SW1573, H1703, H322, H520, HCC4006, and HBEC3-KT (hereon referred as HBEC) cell lines were obtained from the ATCC. A549, H1299, H460, and H727 were maintained in culture no longer than 4 months. Their authenticity was checked by STR profiling. Mycoplasma testing was periodically made. All NSCLC cell lines were cultured at 37 °C in a humidified atmosphere in the presence of 5% CO_2_ and 95% air. Cells were grown in DMEM containing high glucose (4.5 g/L), L-glutamine 4 mM and L-pyruvate 5 mM. The medium was supplemented with 10% FBS and antibiotics (penicillin 100 U/mL and streptomycin 100 µg/mL). HBEC cells were cultured in Keratinocyte-SFM Medium with L-glutamine, EGF, and Bovine Pituitary Extract (Fisher Scientific, Madrid, Spain).

TRC lentiviral pLKO vectors containing human ERK5 and MEK5 shRNA sequences (gene sets RHS4533-EG5598 and RHS4533-EG5607, respectively) were from Dharmacon (Lafayette, Colorado, USA). The specific sequences targeting MEK5 (sh66, 67, 68, and 69) and ERK5 (sh62 and 75) are as follows:

Sh 66 (TRCN0000001466): AGGACCAGTAACCAAGGAGAA

Sh 67 (TRCN0000001467): GCCCTCCAATATGCTAGTAAA

Sh 68 (TRCN0000001468): CCGTTCATCGTGCAGTTCAAT

Sh 69 (TRCN0000001469): CCAATATGCTAGTAAACACAA

Sh 62 (TRCN0000010262): GCTGCCCTGCTCAAGTCTTTG

Sh 75 (TRCN0000010275): GCCAAGTACCATGATCCTGAT

For the production of lentiviruses, host HEK293T cells were co-transfected with four different plasmids obtained from Addgene (Cambridge, MA, USA) using the jetPEI reagent, namely: pLKO vectors, pRSV/Rev, pMDLg/pRRE and pMDG/vsv. All these plasmids were mixed, incubated, and added dropwise to the HEK293T cells as described^15^. 24 h later, the medium containing the recently produced lentiviruses was collected, filtered with 0.45 μm PVDF Millex-HV filters (Merck) and added to the target lung cancer cells together with 6 μg/mL of the polibrene reagent (Sigma Aldrich) in order to increase the transduction efficiency. 8–12 h later, the medium was replaced by fresh medium. After 48 h, positive transduced (knockdown) cells, were selected by adding 3 μg/mL of puromycin (Sigma-Aldrich) to the culture medium for at least 3 days.

### Immunoprecipitation and western blot

Cells were washed twice with PBS and lysed with ice-cold lysis-buffer (140 mM NaCl, 50 mM EDTA, 10% glycerol, 1% Nonidet P-40, 20 mM Tris-HCl, pH 7.0, 1 mM PMSF, 1 mM Na_3_O_4_V, 1 µM pepstatin, 1 µg/mL aprotinin, 1 µg/mL leupeptin, 25 mM β-glycerolphosphate, 10 mM NaF). After centrifugation at 4 °C/15,000 × *g*/10 min, the supernatants were transferred to new tubes and protein concentration measured by the Bradford assay. Immunoprecipitation and western blotting were carried out as described^36^. Briefly, immunoprecipitation was performed by incubating 0.5–3 mg of cell lysate (depending on the specific protein of interest) with the corresponding capture antibody and 60 μL of Protein-A Sepharose CL-4B beads (GE Healthcare, Little Chalfont, England) for a minimum of 2 h at 4 °C. After that, immunocomplexes were pulled down by a quick spin of 15 s at 10,000 × g. The supernatants were then discarded, and the samples washed by adding 1 mL of ice-cold lysis buffer to each sample. This spin and washing proceedings were repeated three times. Immunocomplexes were finally mixed with 40 μL of 2× Sample Buffer (0.05% w/v bromophenol blue; 4% SDS; 20% glycerol; 2.5% β-mercaptoethanol; 100 mM Tris-HCl, pH 6.8) and storage at −20 °C or immediately used.

Samples were resolved by SDS-PAGE and transferred to Inmobilon PVDF membranes (Merck Millipore). The membranes were next blocked by incubation at room temperature for a minimum of 2 h with a TBS-T solution (Tris buffered saline with 0.1% Tween 20; 150 mM NaCl; 20 mM Tris-HCl, pH 7.5) containing 1% BSA or 5% non-fat dried milk to avoid the unspecific binding of antibodies to the membrane. After this step, blots were probed with the appropriate dilution of the primary and secondary antibodies and finally developed with a homemade enhanced chemiluminescence solution (0.08% luminol; 0.02% p-iodophenol; 0.1 M Tris-HCl, pH 9.35) plus 1 μL of 0.44 M H_2_O_2_. All blots or gels derive from the same experiment and were processed in parallel. For western blotting of immunoprecipitated samples, the corresponding loading controls were resolved in a different gel.

### CRISPR assays, xenograft models, and histochemical analysis

NCI-H460 cells were knocked out for ERK5 expression using a *MAPK7* human gene CRISPR/Cas9 knockout kit (reference KN200655) from Origene (Rockville, MD, USA) or MEK5 expression using a MEK5 CRISPR/Cas9 knockout system (references sc-401688 and sc-401688-HDR), and a control CRISPR/Cas9 plasmid (reference sc-418922) from Santa Cruz Biotechnology. ERK5 and MEK5 levels were tested by Western blotting. The proliferation of positive knockout clones was compared to scramble control cells by an MTT assay and three of them (#20 and #57 for ERK5 and #17 for MEK5) were selected for the in vivo experiments.

All animals were manipulated by authorized personal at the animal facility following legal and institutional guidelines. Experimentation was approved by the University of Salamanca Bioethics Committee. For the in vivo studies using ERK5 or MEK5 knockout cells, 25 female BALB/c nude mice (Charles River, Wilmington, MA, USA) were randomly divided into 5 groups of 5 mice each (H460 scramble, #20 or #57 ERK5 CRISPR cells for the ERK5 knockout studies, and H460 scramble or #17 MEK5 CRISPR cells for the MEK5 knockout studies). To assess the effect of the MEK5 or ERK5 knockdown on in vivo tumorigenesis, 10 mice for each cell line (A549, H1299, or H727) were randomly divided into 3 groups (4 mice injected with shControl, 3 mice with shMEK5 and 3 mice with shERK5 cells). 1 × 10^6^ H460 cells, 2.5 × 10^6^ A549 cells, 2.5 × 10^6^ H1299 cells or 3 × 10^6^ H727 cells were injected following standard procedures into the left and right flanks of these mice. Cells were allowed to engraft for one week and measured daily (H460 tumors), three times a week (H1299 and H727 tumors) or twice a week (A549 tumors) until the end of the experiment. Volume progression was calculated by the formula: *V(mm*^*3*^*)* *=* *LxW*^*2*^ *x 0.5*. After animal sacrifice, tumors were resected and divided in two halves. One half was formalin-fixed for histochemical analyses while the other one was immediately frozen in liquid nitrogen. For biochemical analyses, samples were homogenized at a ratio of 1.6 mL of ice-cold lysis buffer supplemented with 1% Triton X-100, per 100 mg of tissue using a Dispomix Drive Homogenizer (Miltenyi Biotec, Bergisch Gladbach, Germany). After centrifugation at 4 °C /15,000 × *g*/20 min, the supernatants were transferred to new tubes and protein concentration measured by the Bradford assay.

For immunohistochemical analyses, mice xenografted tumors were formalin fixed, paraffin embedded, and sectioned. After that, staining with the Ki-67 antibody was performed using the Bond Polymer Refine Detection kit (Leica Biosystems, Newcastle, United Kingdom). Image analyses for the quantitation of Ki67 positive cells were performed using the Leica LAS V3.7 software.

For conducting pharmacological in vivo experiments, 60 mice were xenografted with 1 × 10^6^ H460 cells into the left and right flanks. One week after implantation, mice were divided into 12 groups of 5 mice each, with a mean group tumor volume of 125 mm^3^. BIX02189 and JWG-071 (30 mg/Kg dissolved in 30% 2-hydroxypropyl beta-cyclodextrin, i.p.), and crizotinib (30 mg/Kg in sterile water, p.o.) were administered daily. Cisplatin (3 mg/Kg in physiological saline, i.p.) was given twice per week and pemetrexed (250 mg/Kg in physiological saline, i.p.) once a week. All treatments were administered for 2 weeks. The control group received 30% 2-hydroxypropyl beta-cyclodextrin, i.p. as a vehicle. Standard of care drugs were obtained from the inpatient pharmacy of the University Hospital of Salamanca.

### Patients

Analysis of the relationship between MEK5/ERK5 mRNA expression and outcome (overall survival and post progression survival) of lung cancer patients was carried out using the open access database Kaplan–Meier plotter^37^, available at http://www.kmplot.com. The datasets analyzed as well as the filtering criteria applied to these studies are further described in the “Code availability” section.

Copy Number Alterations (CNA) of *MEK5* and *ERK5* in patients diagnosed with lung cancer were performed from studies available at http://cbioportal.org^38,39^. Specifically, data were collected from the studies with the largest number of patients. These studies also allowed the evaluation of MEK5 and ERK5 mRNA expression levels in correlation with the CNA status of the tumors. In addition, the information corresponding to patient outcome was used to perform the overall survival curves. Patients were stratified into two groups depending on the *MEK5/ERK5* CNA status: those harboring amplification/gain compared to those without such alterations; or patients harboring shallow/deep deletions compared to those without such alterations.

### Quantitative real time-PCR

RNA from NSCLC cells was isolated using the RNeasy mini kit (Qiagen, CA, USA). cDNA was synthesized with M-MLV reverse transcriptase (Promega, Madison, WI, USA). Expression levels were determined by qRT-PCR with the primers: Forward: 5′-ACGTGAAGCCCTCCAATATG-3′ and reverse: 5′-ACTGCTCCCCTGAAATCCTT-3′ for *MEK5*; Forward: 5′-TGCACCACCAACTGCTTAGC-3′ and reverse 5′-GGCATGGACTGTGGTCATGAG-3′ for GAPDH, which was used as control. The reactions were performed using iQTM SYBR Green Supermix (Bio-Rad Laboratories, Madrid, Spain). Increased fluorescence was measured and recorded using the Bio-Rad iQ5 software. The results were normalized to the reference gene, *GAPDH*. The relative quantitation of gene expression was performed through the cycle threshold (CT) increment method^40^.

### Cell proliferation assays

Cells were plated in 24-well plates in quadruplicates at 7000 cells (A549 and H1299), 5000 cells (H460), 12000 cells (H727), and 4500 cells (HBEC) per well, in medium containing or not the appropriate drugs and incubated for the indicated periods of time. Cell proliferation was analyzed by an MTT-based assay as previously described^36^. Briefly, the MTT reagent (Sigma Aldrich) was dissolved in PBS and added to the culture medium to a final concentration of 0.5 mg/mL. Cells were then incubated at 37 °C for 1 h in the dark. After that, the medium was aspirated and 500 μL of DMSO were immediately added to each well, and plates were incubated in an orbital shaker for 10 min in the dark to facilitate the dissolution of the formazan crystals. Finally, the absorbance was read at 570 nm using an ULTRA Tecan plate reader (Tecan; Männedorf, Switzerland). The results were represented either as mean absorbance ± SD of the quadruplicates or relativized as percentage from mean absorbance of the control (untreated or scramble) condition of an experiment that was repeated two or three times. Alternatively, proliferation was evaluated by direct cell counting. In those cases, cells were similarly cultured in duplicates in 6-well plates. At the indicated times, cells were trypsinized and diluted in a 1/10 or 1/20 solution of ISOTON (Beckman Coulter, Brea, CA, USA). Finally, the solution containing the individualized cells was counted using a Z1 Coulter Particle Counter (Beckman Coulter).

### Cell cycle and apoptosis assays

Cell cycle profiling was carried out in asynchronous as well as in synchronized cultures by treatment with nocodazole (121 ng/mL, 18 h). Cells were treated or not with BIX02189 5 µM for the indicated times. DNA content and cell cycle analyses were performed as described^41^. Briefly, cells were trypsinized, collected, gently washed with PBS, resuspended in ice-cold 70% ethanol, and incubated at −20 °C overnight for permeabilization. The processed cells were washed again and resuspended in 1 mL of PBS containing 50 µg of PI (Propidium Iodide) and 200 µg of DNase-free RNase A (Sigma Aldrich). After incubation at room temperature in the dark for 1–2 h, DNA content was measured using an Accuri C6 flow cytometer (BD Biosciences). The appropriate gating, debris exclusion, and analyses of the percentage of cells in the different phases of the cell cycle were performed using the C6 software (BD Biosciences).

The BrdU incorporation assay was carried out to determine the cells undergoing DNA synthesis. To that end, 10 µM BrdU was directly added into cell culture media and incubated for 45 min. After that, cells were fixed, permeabilized, treated with DNase, and stained using a FITC BrdU Flow Kit according to the provider’s instructions (BD Biosciences). 20 µL of 7-AAD were added to stain total DNA and the fluorescence was read in an Accuri C6 flow cytometer.

Apoptosis analysis and caspase activity assays were performed as previously described^42^ after treatment of cells with BIX02189 5 µM for the indicated times. Briefly, H460 cells were trypsinized, collected, washed twice with cold PBS and resuspended in 20 µL of cold binding buffer (10 mM HEPES, 140 mM NaCl, and 2.5 mM CaCl_2_, pH 7.4). Cells were then incubated with 5 µL of Annexin V/FITC and 5 µL of 50 µg/mL propidium iodide for 15 min in the dark. After that, another 400 µL of binding buffer were added to labeled cells, which were immediately read in an Accuri C6 flow cytometer. Results were analyzed with the C6 software.

Alternatively, caspase activity assays were performed. Cells were treated with the appropriate drug and lysed in lysis buffer. 50 µg of cell lysates were placed in three different wells (triplicates) of 96-well plates. The final volume of the lysates was taken to 100 µL by 1× Caspase Buffer (25 mM HEPES pH 7.4, 150 mM NaCl, 1 mM EDTA, 0.1% CHAPS, 10% sucrose). 100 µL of 2× Caspase Reaction Buffer supplemented with 20 mM DTT and 10 µM fluorescent labeled caspase substrate Ac-IETD-AFC (caspase 8) or Ac-DEVD-AFC (caspase 3) were added to each well containing cell lysates. The plate was shaken to mix the solution and incubated at 37 °C in the dark for 1 h. The signals were measured at 400/505 nm in a multi-well fluorescent reader from BioTek (Winooski, VT, USA).

### Statistical analyses

For in vitro studies, the analyses of variance between variables were calculated for each experiment according to the Fisher’s exact test. Unless otherwise indicated, comparison of continuous variables between groups were carried out using a two-sided Student’s *t*-test. For the in vivo tumor growth experiments, normality of data distribution was analyzed by the Kolmogorov-Smirnov test. One‐way ANOVA followed by Bonferroni multiple comparison test was used to compare control group versus treatment groups. Wilcoxon–Mann–Whitney test was used to compare two groups when the distribution was not assumed to be normal. Mean differences between groups were considered statistically significant at *p*-value ≤ 0.05 (*), *p* ≤ 0.01 (**) or *p* ≤ 0.001 (***). Statistical analyses were performed using SPSS 19.0 software (SPSS Inc., Chicago, IL, USA). Additional information is described in the appropriate figure legends.

### Reporting summary

Further information on research design is available in the Nature Research Reporting Summary linked to this article.

## Supplementary information

Supplementary Information

Reporting Summary

## Data Availability

The authors declare that relevant data supporting the findings of this study are available within the paper and its Supplementary information files.

## Code availability

The KM-plotter studies were performed using the multigene classifier tool by selecting the mean expression values for both MEK5 (*MAP2K5* gene, Affymetrix probe id 211370_s_at) and ERK5 (*MAPK7* gene, Affymetrix probe id 35617_at) on the 2015 version of the database. The gene expression values of MEK5 and ERK5 were computed together and the cut off values used to split patients into low or high MEK5 or ERK5 expression were automatically selected by the database for each cohort of patients by utilizing the “Auto select best cutoff” option. These cut off values were those bringing the most significant *p*-value between the two groups, which were scored computing one by one all the possible percentiles between the lower and upper quartiles. The “Censore at threshold” option was also selected. The patient cohorts selected for the studies (lung cancer, adenocarcinoma, squamous carcinoma, smokers, non-smokers) as well as the survival option analyzed (overall survival or post-progression survival) are indicated in the appropriate figures. Analyses were not filtered by tumor grade, tumor stage, gender, surgery’s success nor chemotherapy/radiotherapy treatments. The array quality controls excluded biased arrays. Cox regression was used to compute the Hazard Ratio and *p*-value. False discovery rates are indicated. The cBioportal studies were performed on the lung cancer datasets containing the largest number of samples and patient survival data: Lung Adenocarcinoma (TCGA, PanCancer Atlas) and Lung Squamous Cell Carcinoma (TCGA, PanCancer Atlas). The analyses of copy number alterations in MEK5 and ERK5 were performed by selecting the “Putative copy-number alterations from GISTIC” option. The evaluation of amplifications or gains were carried out by introducing the Onco Query Language command “MAP2K5: AMP GAIN” and “MAPK7: AMP GAIN”, respectively. In parallel, the evaluation of shallow or deep delteions were carried out by introducing the Onco Query Language command “MAP2K5: HOMDEL HETLOSS” and “MAPK7: HOMDEL HETLOSS”. The profile selected for the analyses of the mRNA expression levels was “mRNA expression z-scores relative to diploid samples (RNA Seq V2 RSEM)”.
