# Supplementary material for: Clinical, genetic and pharmacological data support targeting the MEK5/ERK5 module in lung cancer
Source: NPJ Precis Oncol. 2021 Aug 17;5:78. doi: 10.1038/s41698-021-00218-8 (PMC8371118; doi:10.1038/s41698-021-00218-8)

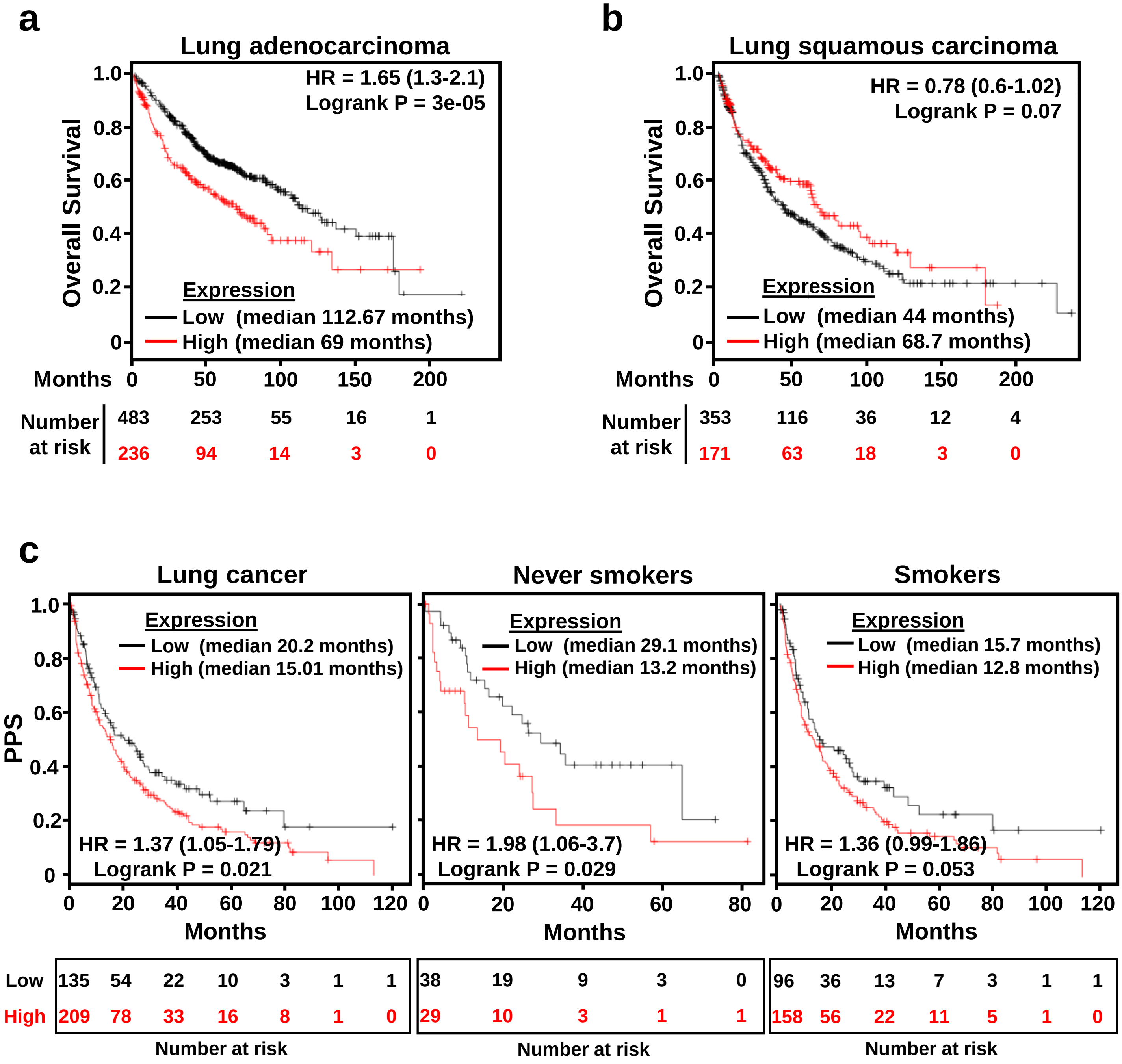


**Supplementary Figure 1.** Kaplan-Meier analyses of the relationship between MEK5/ERK5 expression and Overall Survival in (a) lung adenocarcinoma (n=719), or (b) squamous cell carcinoma (n=524) patients. Data were collected from the Kaplan-Meier plotter database. The p-value, hazard ratio, median survival and number of patients at risk are indicated. FDR values for the analyses of the lung adenocarcinoma and lung squamous carcinoma cohorts are 0.01 and 1, respectively. c) Kaplan-Meier analyses of the relationship between MEK5/ERK5 expression and Post Progression Survival (PPS) in lung cancer (n=344, left panel), never-smoker (n=67, central panel) and those excluding never-smoker (n=254, right panel) patients collected in the public Kaplan-Meier plotter database. The p-value, hazard ratio, median survival and number of patients at risk are indicated. FDR values for the analyses of the whole lung cancer, never smokers and smokers cohorts are 0.50, 0.50, and 1, respectively.

# Supplementary Figure 1

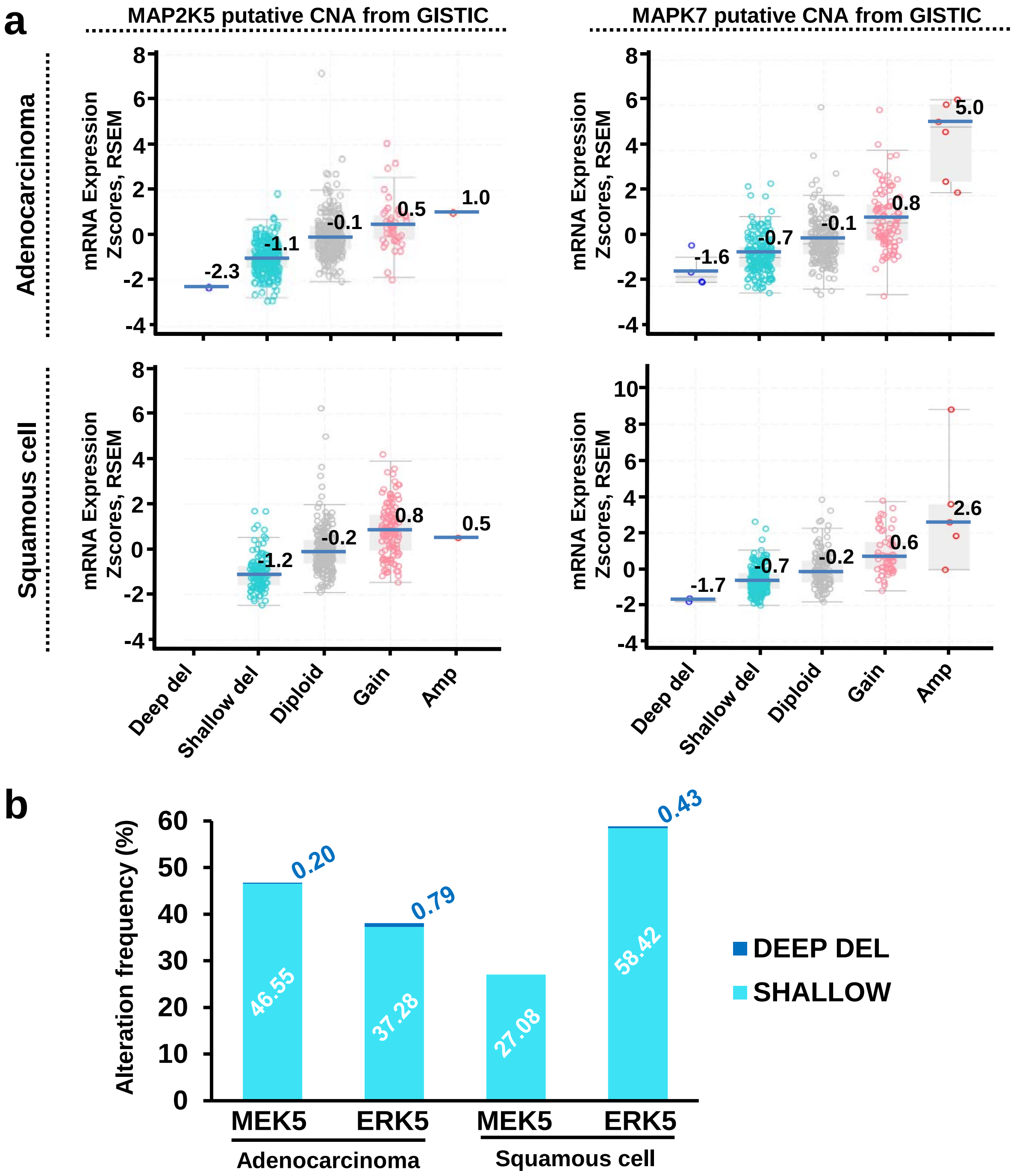


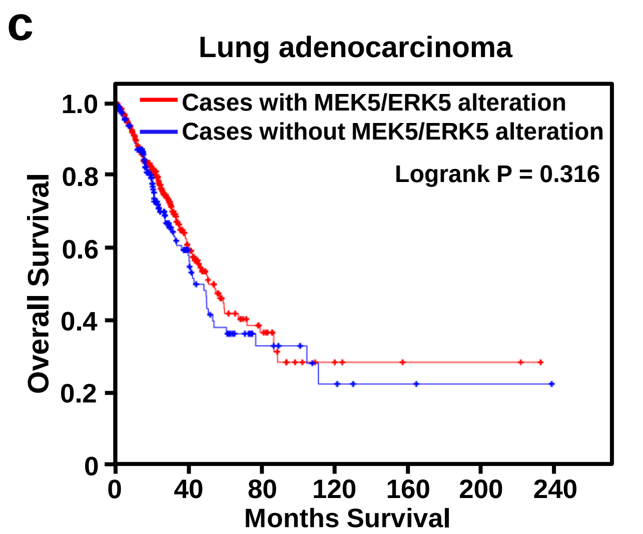


| Cases, Total | Deceased | Median OS |
|---|---|---|
| 309 | 106 | 53.65 months |
| 185 | 70 | 47.80 months |

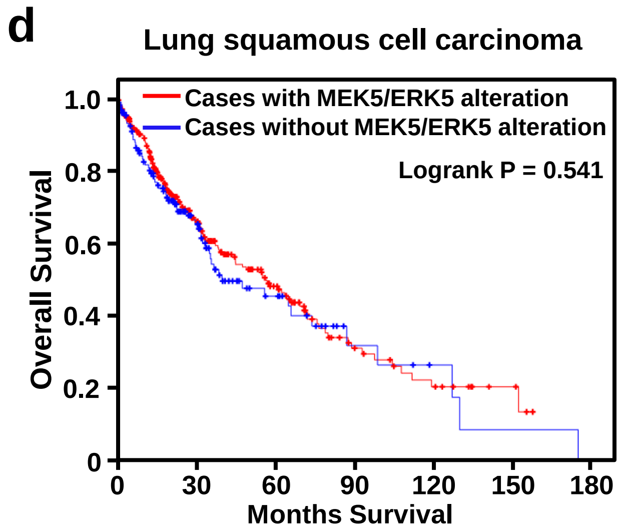


| Cases, Total | Deceased | Median OS |
|---|---|---|
| 316 | 137 | 56.32 months |
| 147 | 63 | 46.78 months |

**Supplementary Figure 2**

**Supplementary Figure 2.** a) Relationship between Copy Number Alterations (CNA) of *MEK5* or *ERK5* genes and the mRNA expression of patients collected in the lung adenocarcinoma or lung squamous cell carcinoma studies from cBioPortal (TCGA Pan-Lung cancer Study). The blue lines correspond to the median mRNA expression value of each CNA subgroup. The copy number data sets were generated by the GISTIC algorithm. b) The cBioPortal database (TCGA PanCancer Atlas study) was explored to determine the deletions present in *MEK5* and *ERK5* genes of lung adenocarcinoma (n=503) and lung squamous cell carcinoma (n=469) patients. Patients with deep or shallow deletions are represented as percentage from the total patients included in the study. c) and d) Overall survival curves of lung adenocarcinoma (n=494) and lung squamous carcinoma (n=463) patients with available clinical data from patients harboring shallow or deep deletion in *MEK5* or *ERK5* (red line), compared to those without such alterations (blue line). The *p*-value of the studies, follow-up (months) and median overall survival are indicated.

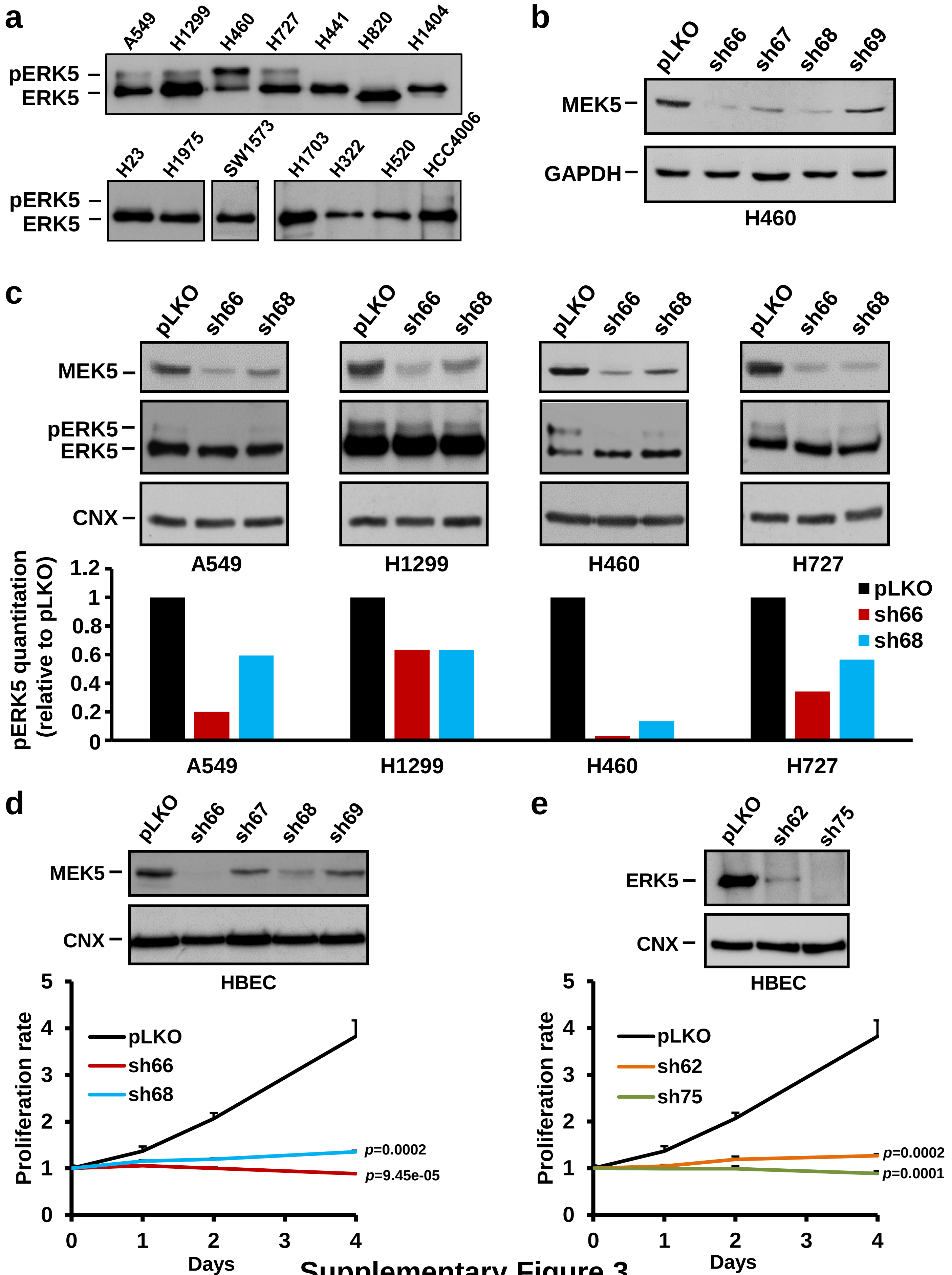


Supplementary Figure 3

**Supplementary Figure 3.** a) Expression of ERK5 and pERK5 in a panel of 14 NSCLC cell lines. Cell extracts were prepared and 1 mg immunoprecipitated with the anti-ERK5 antibody and Western blots probed with the same antibody. b) H460 cells were infected with a scramble control sequence or 4 different MEK5 specific shRNA sequences. Cells were lysed and 70 µg of cell extracts were used to determine the MEK5 knockdown by Western blotting with the anti-MEK5 antibody. GAPDH was used as loading control. c) Cells infected with pLKO shControl, sh68 and sh66 were lysed and MEK5 levels were evaluated on 70 micrograms of cell extracts by Western blotting with the anti-MEK5 antibody. The effect of MEK5 knockdown on ERK5 activation was analyzed by immunoprecipitating 1 mg of protein followed by Western blotting with the anti-ERK5 antibody. The bar graph shows the quantitation of the pERK5 levels, relativized to the levels present in the pLKO-infected cells. Calnexin was used as loading control. d) Effect of sh66 and sh68 MEK5 targeting shRNA sequences on HBEC proliferation. 4500 cells were plated and proliferation was measured at the indicated times by MTT proliferation assays. The Western blot at the top shows the effect of different knockdown sequences on the levels of MEK5. e) Effect of sh62 and sh75 ERK5 targeting shRNA sequences on HBEC proliferation. The Western blot at the top shows the effect of these knockdown sequences on the levels of ERK5. Note that the proliferation of pLKO is the same in panels d) and e) since the results plotted come from the same experiment.

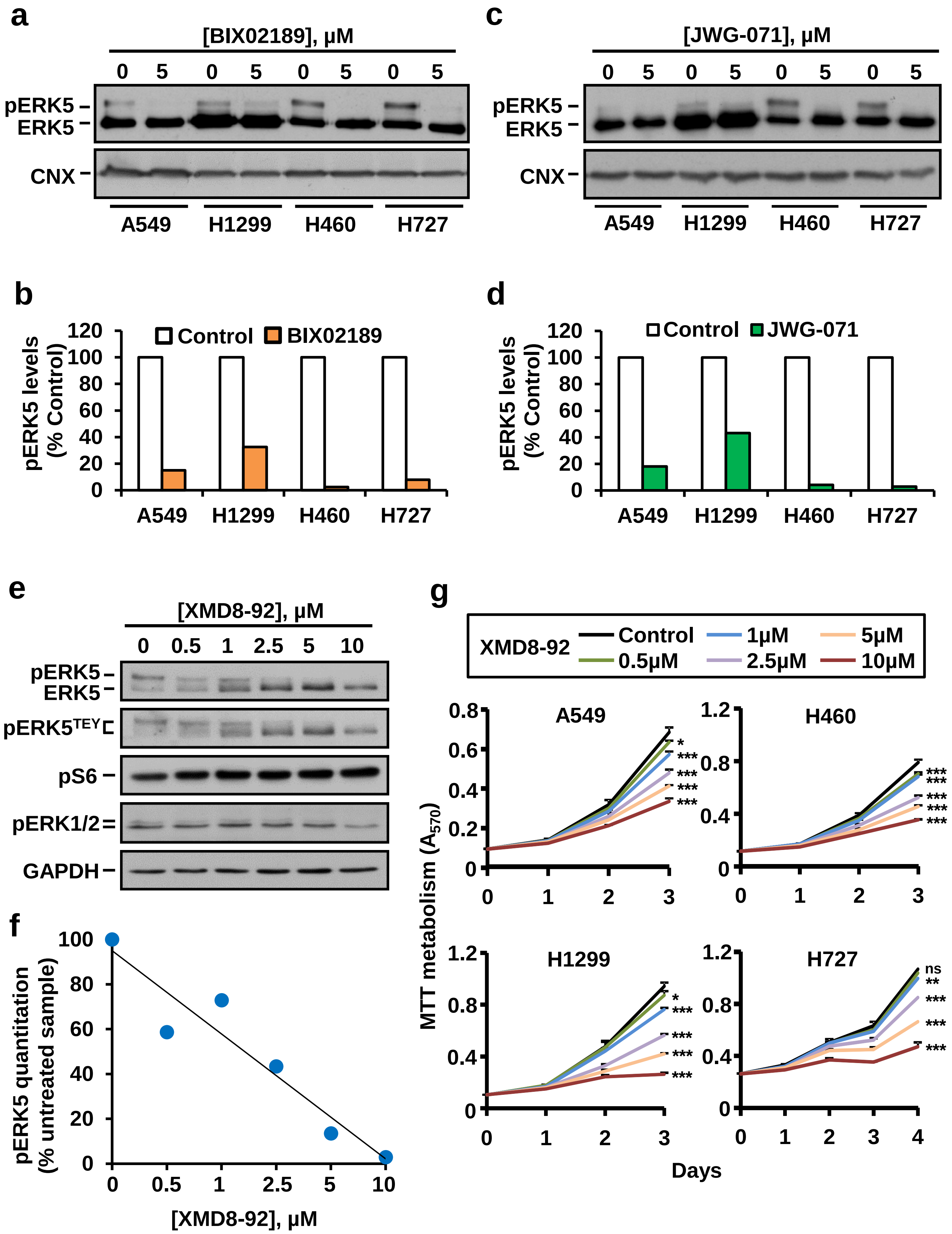


Supplementary Figure 4

**Supplementary Figure 4.** a) NSCLC cell lines were treated with BIX02189 5 μM for 7 hours and the status of ERK5 phosphorylation analyzed by immunoprecipitation and Western blotting with the anti-ERK5 antibody. b) pERK5 levels (upper band) from (a) were quantified using the ImageJ software and represented as percentage from control untreated cells. c) NSCLC cell lines were treated with JWG-071 5 μM for 7 hours and the status of ERK5 phosphorylation was analyzed by immunoprecipitation and Western blotting with the anti-ERK5 antibody. d) pERK5 levels (upper band) from (c) were quantified using the ImageJ software and represented as percentage from control untreated cells. e) H460 cells were treated with increasing doses of XMD8-92 for 48 hours. Inhibition of ERK5 activation was evaluated by immunoprecipitation with the anti-ERK5 antibody and probed with the C terminal anti-ERK5 or anti-pERK5TEY antibodies. pS6 and pERK1/2 were used as controls to assure that XMD8-92 did not affect the PI3K and ERK1/2 routes. GAPDH was used as loading control. f) Quantitation of pERK5 band in H460 cells from (e) using the ImageJ software. Data represents the percentage of the upper pERK5 band after XMD8-92 treatment with respect to such band in control H460 untreated cells. g) NSCLC cells were plated in 24-well dishes and treated with increasing doses of XMD8-92. Cell proliferation was measured at the indicated times by MTT. Results are expressed as mean ± SD of an experiment that was repeated three times. *, $p \leq 0.05$; **, $p \leq 0.01$; ***, $p \leq 0.001$. Exact *p*-values are shown in Supplementary Table 1c.

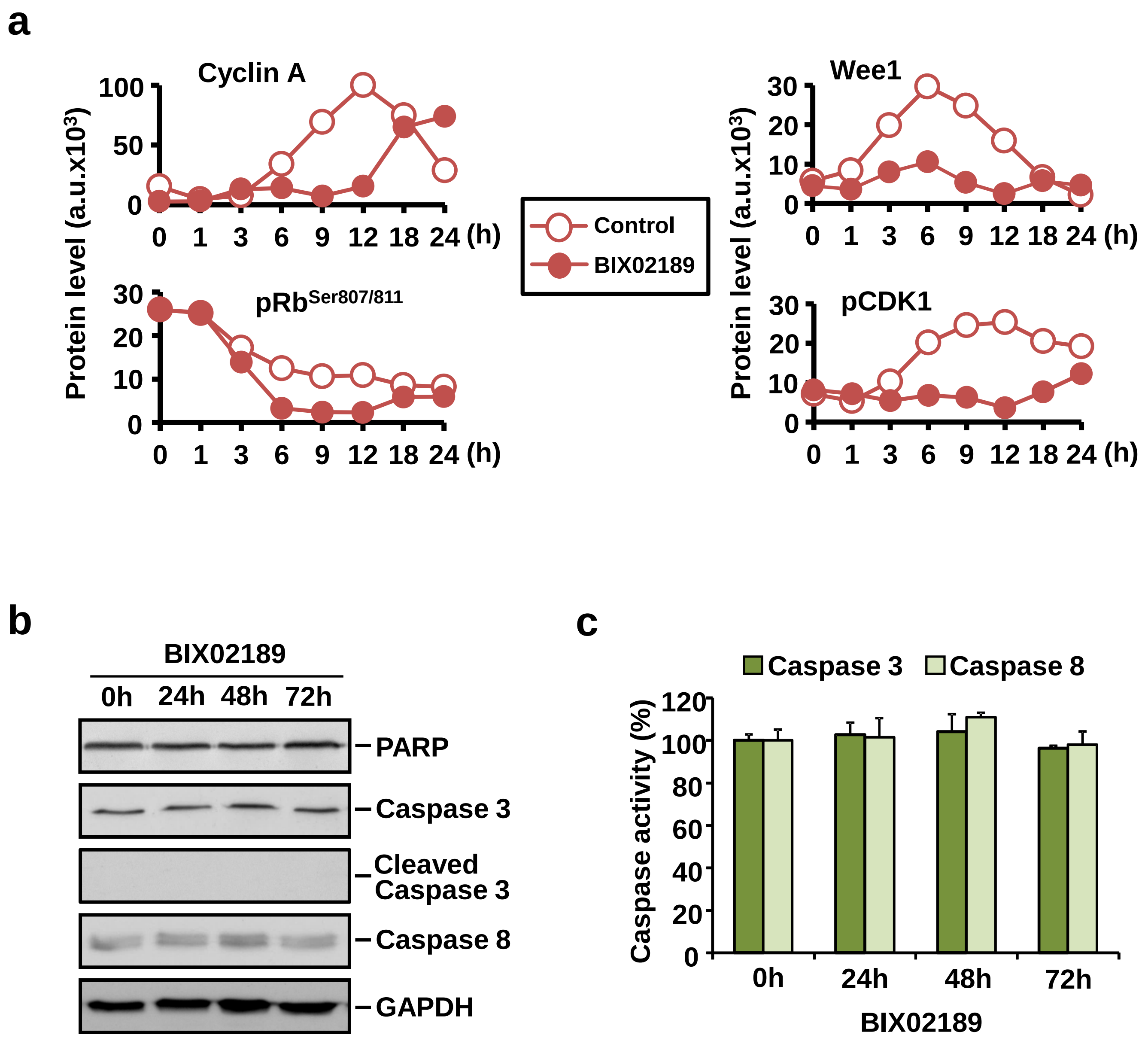


**Supplementary Figure 5.** a) Cyclin A, Wee1, pRb$^{Ser807/811}$ and pCDK1 levels from blots included in Figure 5E were quantified using the ImageJ software. b) Biochemical analyses of PARP, caspase 3, cleaved caspase 3 and caspase 8 by Western blotting after 0, 24, 48 or 72 hours of BIX02189 5 μM treatment in the H460 cell line. c) Caspase 3 and caspase 8 activity assays performed at the indicated times after BIX02189 5 μM treatment. Histograms show the mean ± SD of an experiment that was repeated twice.

# Supplementary Figure 5

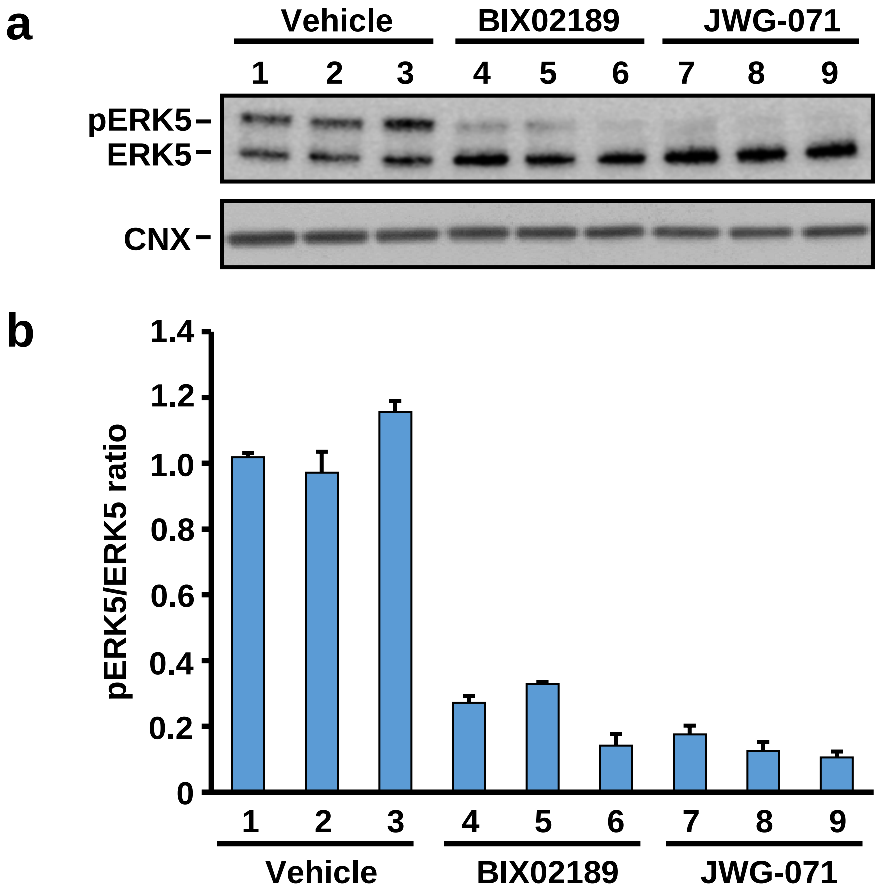


**Supplementary Figure 6.** a) pERK5 and ERK5 levels in tumors (n=3 per condition) from mice treated with vehicle, BIX02189 or JWG-071. Samples were analyzed by immunoprecipitating 1 mg of protein followed by Western blotting with the anti-ERK5 antibody. Calnexin was used as loading control. b) Quantitative analysis of pERK5/ERK5 ratios in tumors from mice xenografted with H460 cells and treated as indicated. Westerns (n=2) were quantitated using the Image Lab software, and the results represented mean ± SD.

# Supplementary Figure 6

**a**

| [BIX], µM | 0.5 | 1 | 2.5 | 5 | 10 |
|---|---|---|---|---|---|
| **A549** | 0.0436 | 0.0152 | 0.0041 | 0.0004 | 1.55E-06 |
| **H1299** | 0.0980 | 0.0704 | 0.0069 | 6.84E-06 | 1.45E-06 |
| **H460** | 0.1752 | 0.0065 | 3.78E-06 | 7.07E-06 | 5.49E-10 |
| **H727** | 0.0077 | 0.0112 | 0.0002 | 4.94E-06 | 2.23E-07 |

**b**

| [JWG], µM | 0.5 | 1 | 2.5 | 5 | 10 |
|---|---|---|---|---|---|
| **A549** | 0.0402 | 0.0010 | 1.08E-05 | 0.0001 | 6.26E-05 |
| **H1299** | 0.0023 | 0.0008 | 2.35E-05 | 5.70E-07 | 5.76E-05 |
| **H460** | 0.0684 | 2.77E-06 | 1.02E-08 | 0.0001 | 1.05E-08 |
| **H727** | 0.0076 | 0.0022 | 5.67E-06 | 3.17E-07 | 1.54E-08 |

**c**

| [XMD], µM | 0.5 | 1 | 2.5 | 5 | 10 |
|---|---|---|---|---|---|
| **A549** | 0.0233 | 0.0002 | 7.15E-06 | 0,0001307 | 2.81E-07 |
| **H1299** | 0.0178 | 2.08E-05 | 2.13E-07 | 2.40E-05 | 7.77E-09 |
| **H460** | 0.0004 | 0.0006 | 8.75E-07 | 1.40E-07 | 1.67E-08 |
| **H727** | 0.3643 | 0.0067 | 1.46E-05 | 2.48E-07 | 9.27E-08 |

**d**

| $G_0/G_1$ | S | $G_2/M$ |
|---|---|---|
| 0.0015 | 0.04718 | 0.0077 |

**e**

| | BIX | JWG | Crizo | Cis | Peme | BIX-Crizo | BIX-Cis | BIX-Peme | JWG-Crizo | JWG-Cis | JWG-Peme |
|---|---|---|---|---|---|---|---|---|---|---|---|
| **Control** | 0.0072 | 0.019 | 0.0072 | 0.0002 | 0.0935 | 0.0106 | <0.0001 | 0.0259 | 0.0102 | <0.0001 | 0.0251 |

**f**

| | BIX | JWG | Crizo | Cis | Peme | Bix-Crizo | Bix-Cis | Bix-Peme | JWG-Crizo | JWG-Cis | JWG-Peme |
|---|---|---|---|---|---|---|---|---|---|---|---|
| **Tumor growth inhibition (% control)** | 53.19 | 48.72 | 53.20 | 67.67 | 40.62 | 51.46 | 90.37 | 47.22 | 51.63 | 82.85 | 47.37 |

**g**

| | BIX-Crizo | Bix-Cis | Bix-Peme | JWG-Crizo | JWG-Cis | JWG-Peme |
|---|---|---|---|---|---|---|
| **BIX** | 0.7394 | 0.0009 | 0.2799 | | | |
| **JWG** | | | | 0.9705 | 0.0021 | 0.7959 |
| **Crizo** | 0.5787 | | | 0.5787 | | |
| **Cis** | | <0.0001 | | | 0.0052 | |
| **Peme** | | | 0.9118 | | | 0.6305 |

**Supplementary Table 1.** a-c) Exact *p*-values (Fisher's exact test followed by two-sided Students's *t* test) for the experiments shown in Fig. 4c (panel a), 4f (panel b), and Supplementary Fig. 4g (panel c). d) Exact *p*-values (Fisher's exact test followed by two-sided Students's *t* test) for the experiment shown in Fig. 5a. e) Exact *p*-values (one-way ANOVA followed by Bonferroni multiple comparison test) for comparison of mean tumor volumes between untreated and treated mice groups shown in Fig. 6b and c. f) Tumor growth inhibition, measured as percentage from control untreated mice group, provoked by the different drug treatments shown in Fig. 6b and c. g) Exact *p*-values (Wilcoxon-Mann-Whitney test) for comparison of mean tumor volumes between individual and combined drug treatments of mice groups shown in Fig. 6b and c.

# Supplementary Table 1

Figure 2b

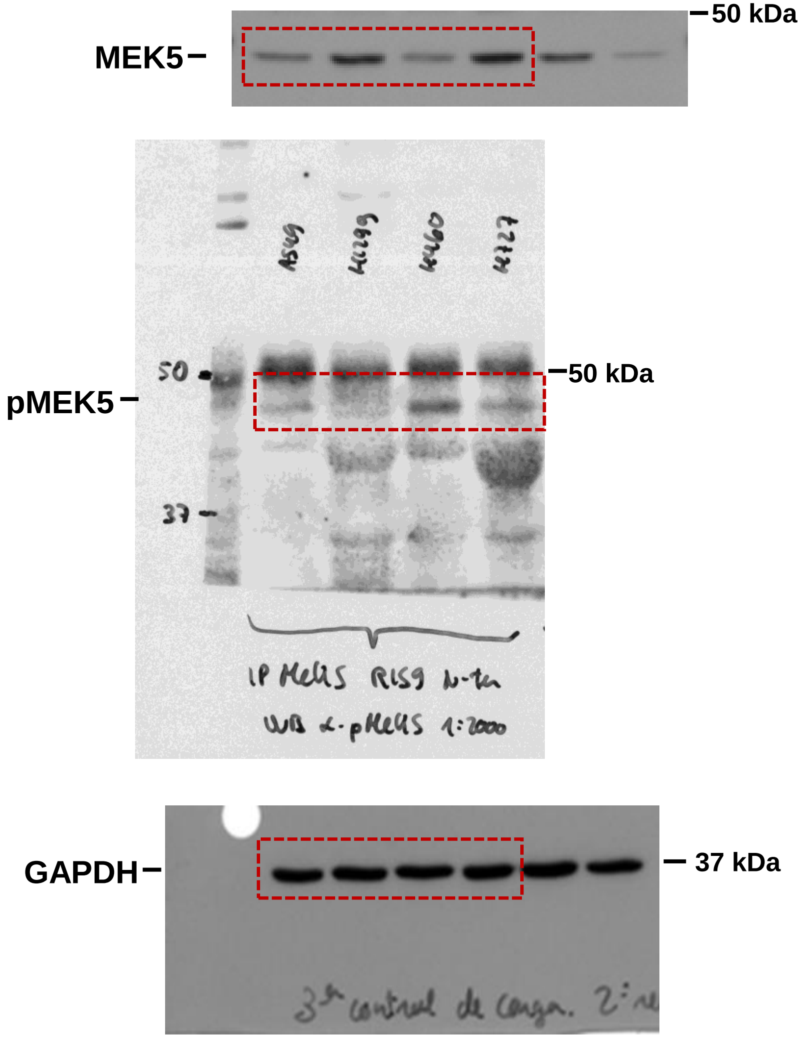


Figure 2c

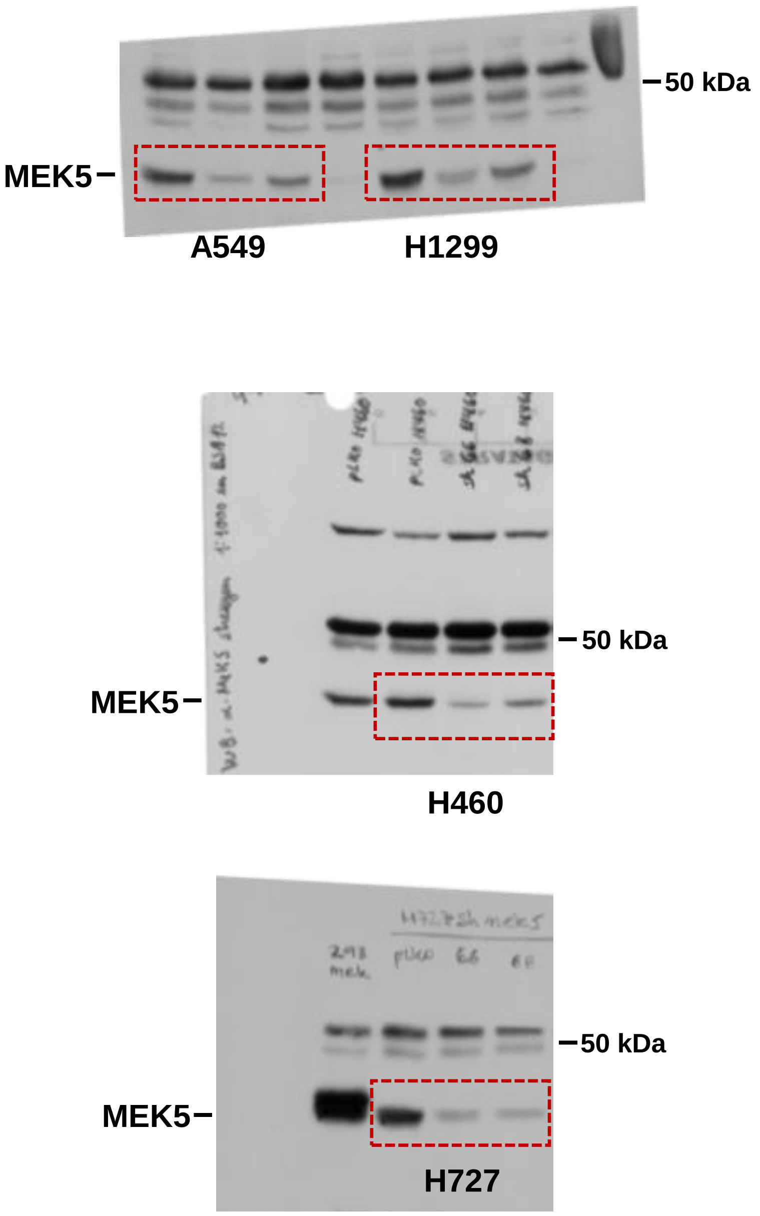

Figure 2d

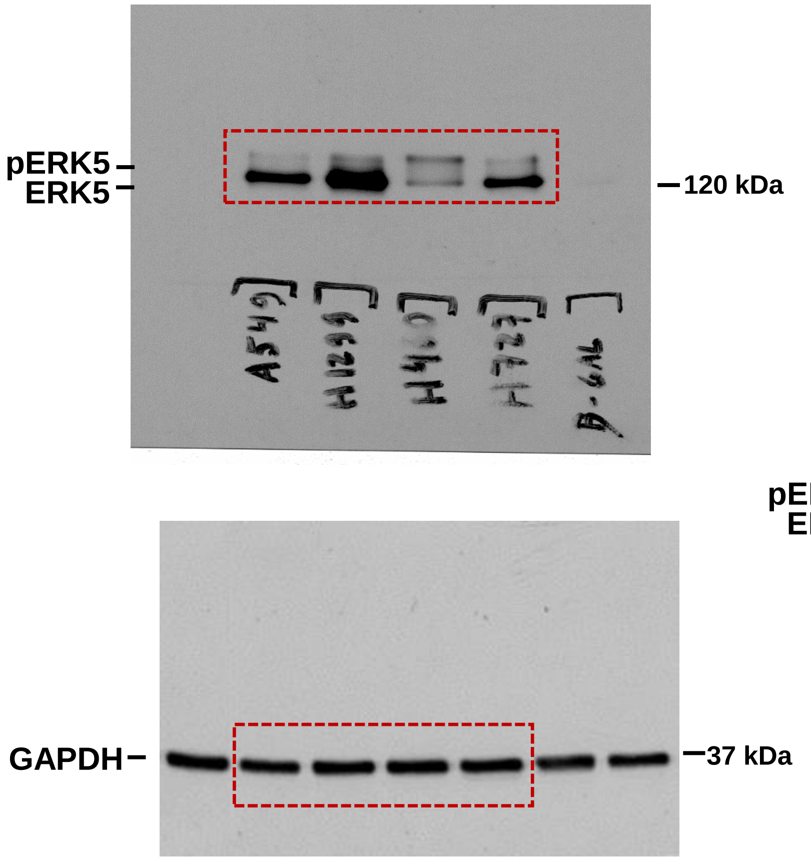


Figure 2f

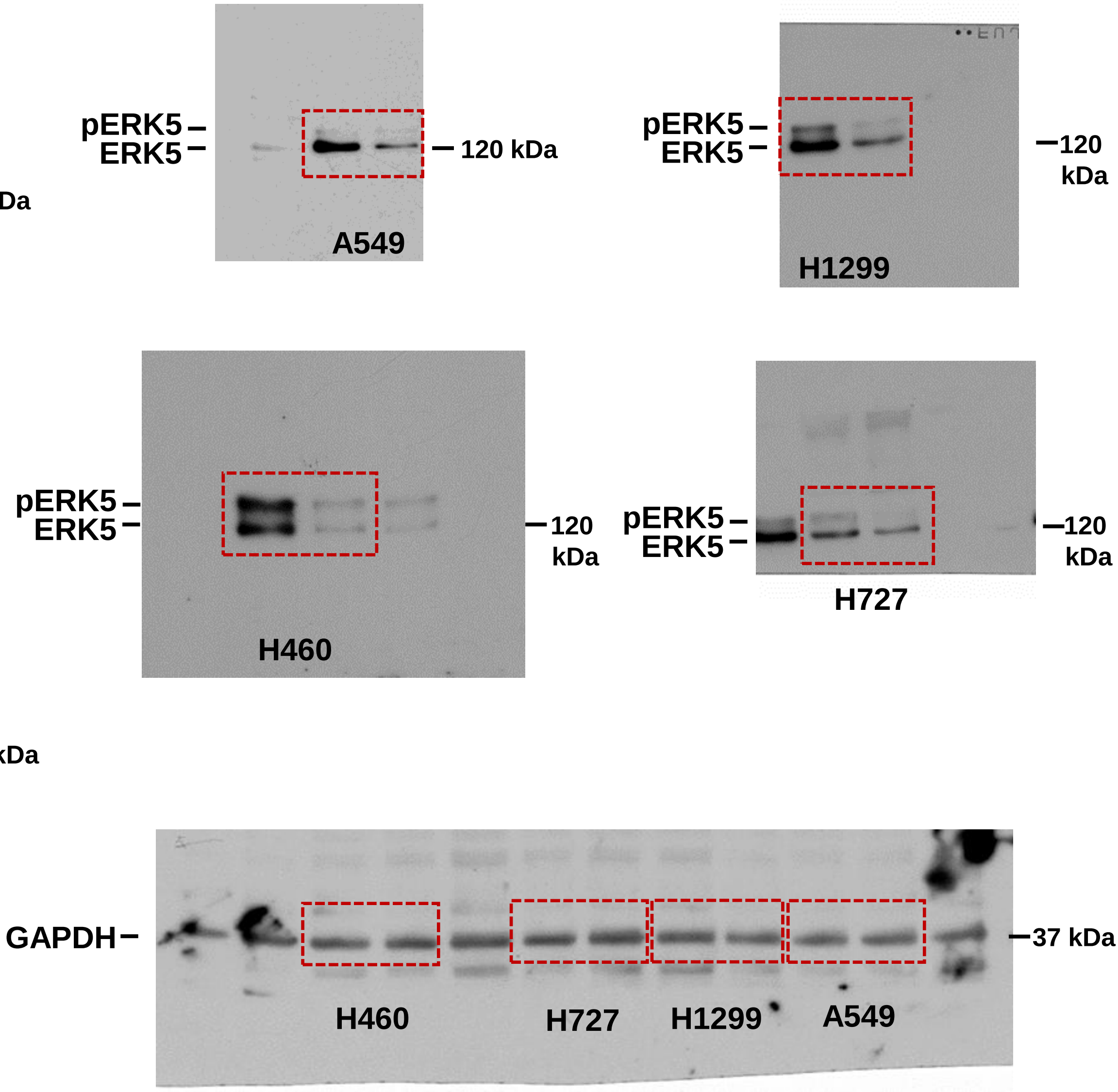

Figure 3a

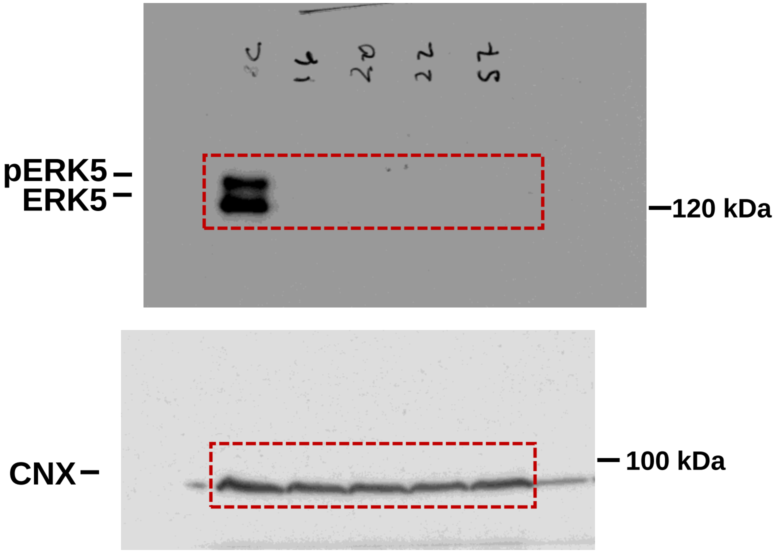


Figure 3c

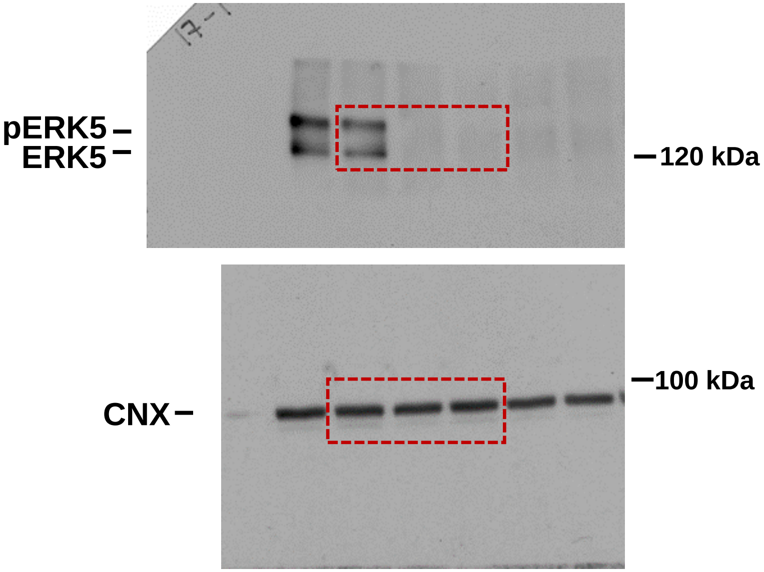


Figure 3f

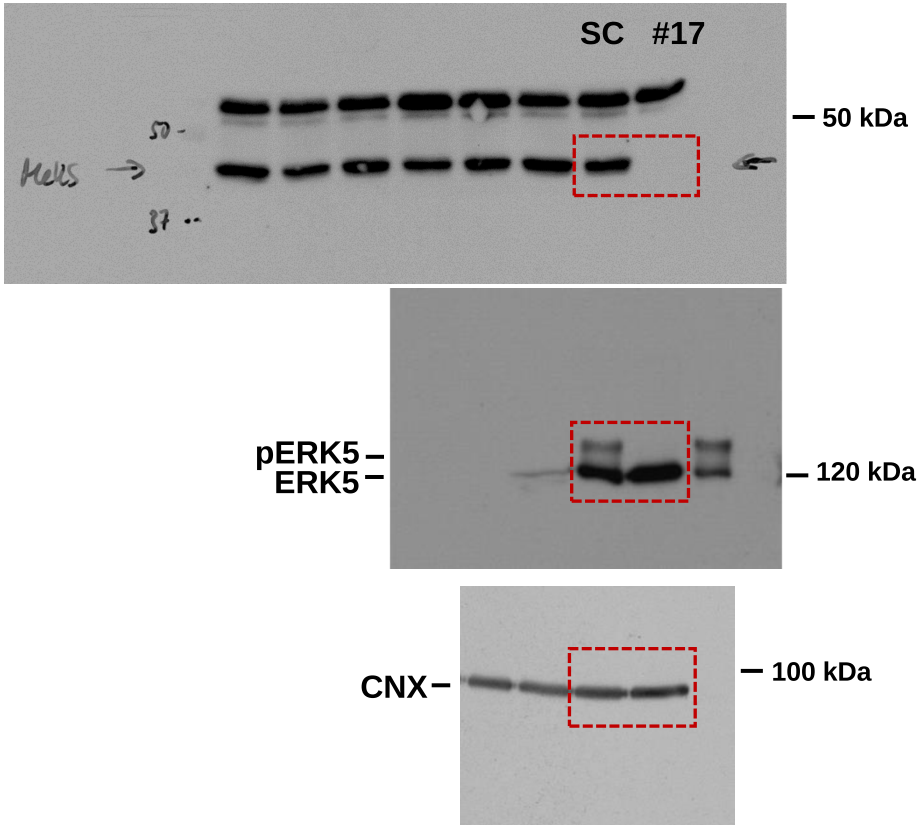

## Figure 4a

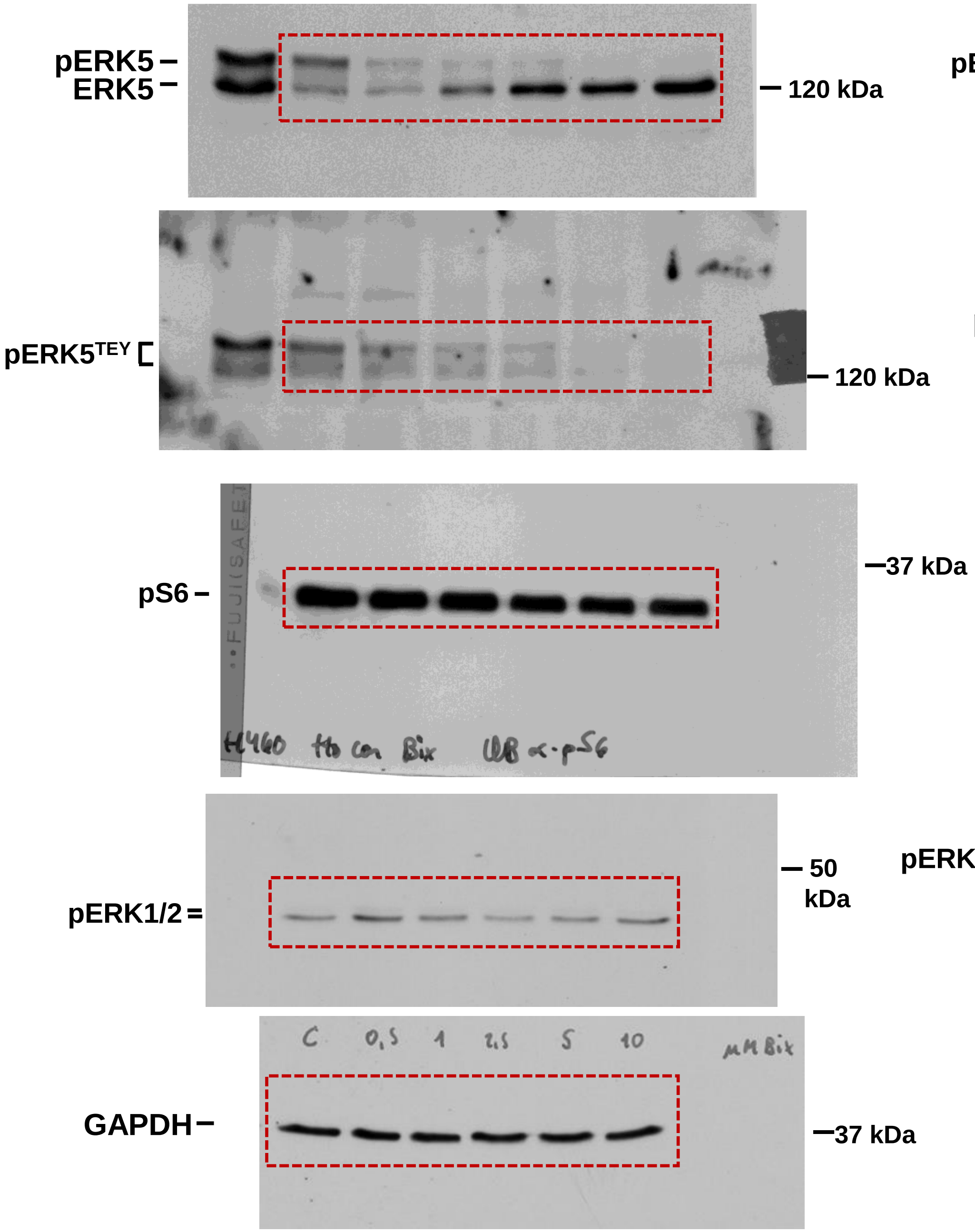


## Figure 4d

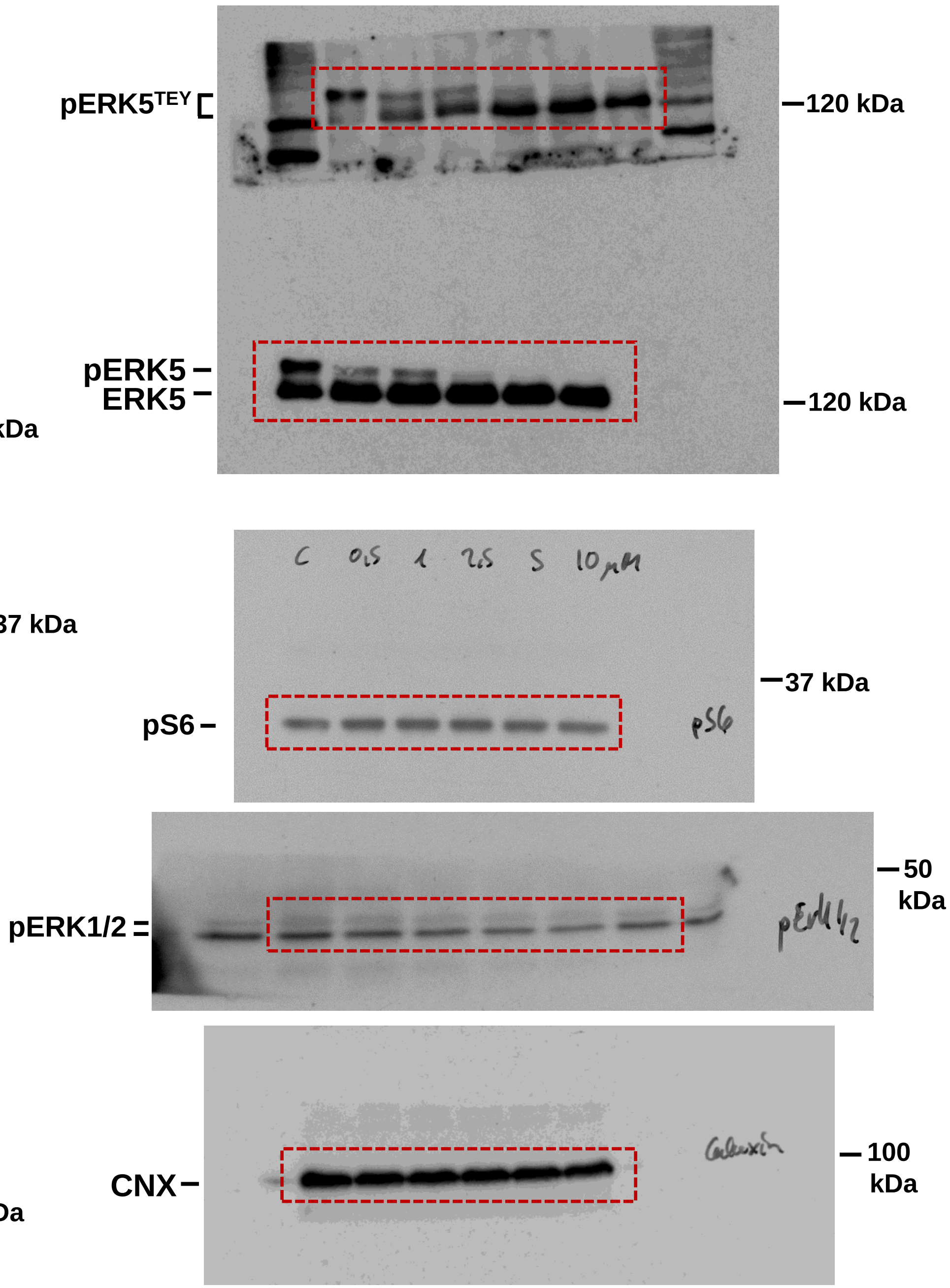

Figure 5e

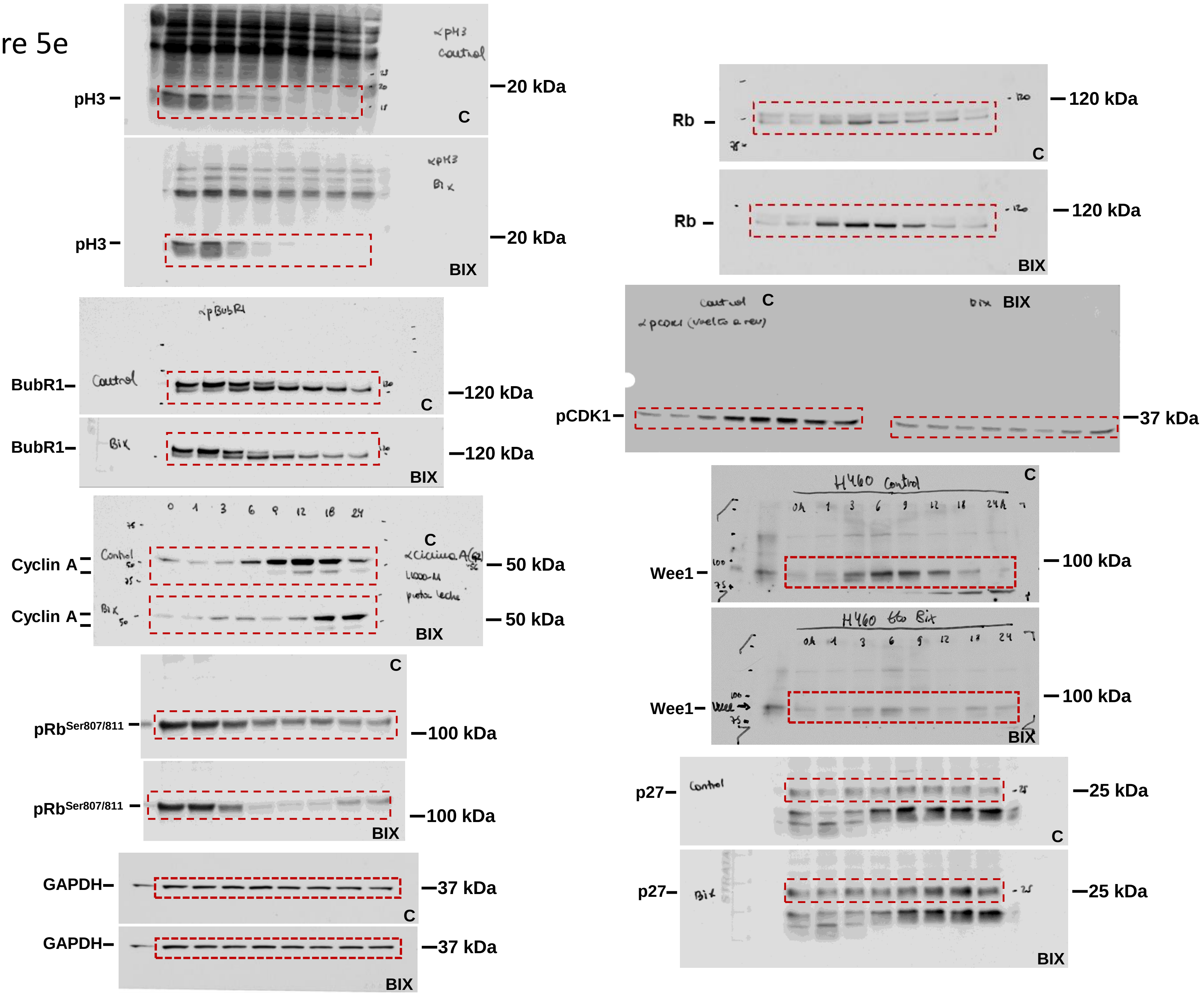

pH3
20 kDa
C
pH3
20 kDa
BIX
BubR1
120 kDa
C
BubR1
120 kDa
BIX
Cyclin A
50 kDa
C
Cyclin A
50 kDa
BIX
C
pRbSer807/811
100 kDa
pRbSer807/811
100 kDa
BIX
GAPDH
37 kDa
C
GAPDH
37 kDa
BIX
Rb
120 kDa
C
Rb
120 kDa
BIX
C
BIX
pCDK1
37 kDa
C
Wee1
100 kDa
Wee1
100 kDa
BIX
p27
25 kDa
C
p27
25 kDa
BIX

## Supplementary Figure 3a

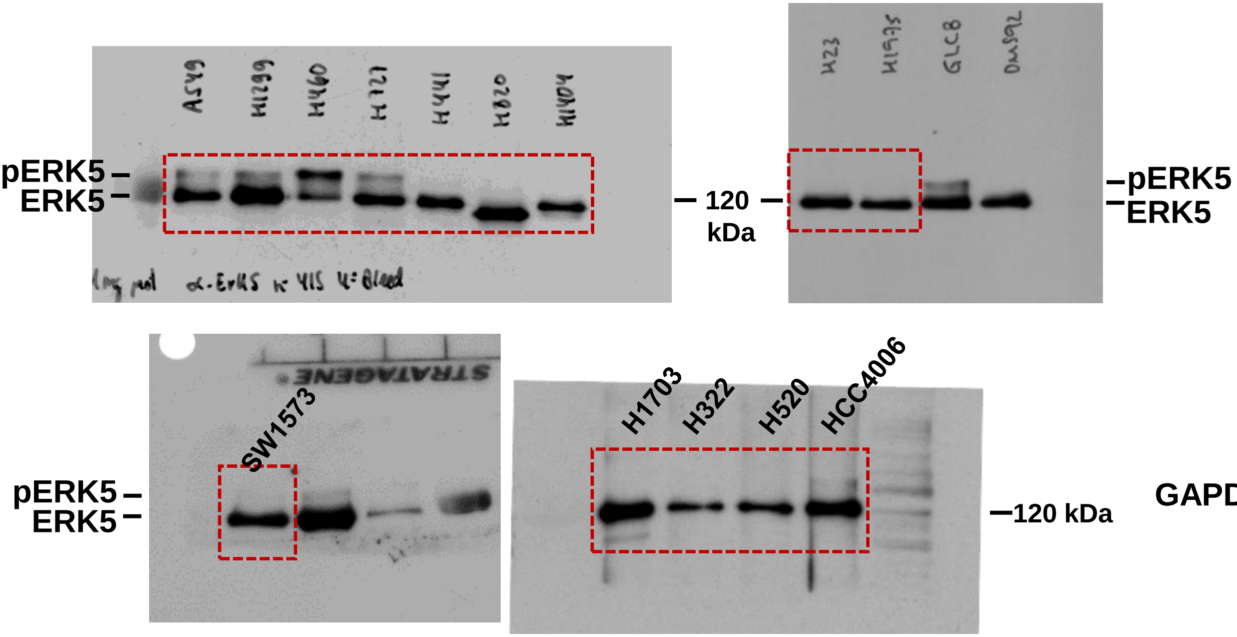


## Supplementary Figure 3b

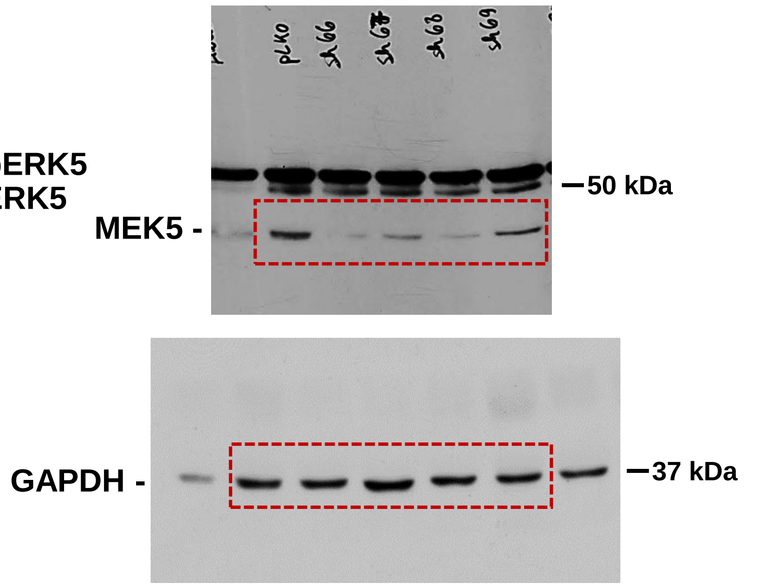


## Supplementary Figure 3c

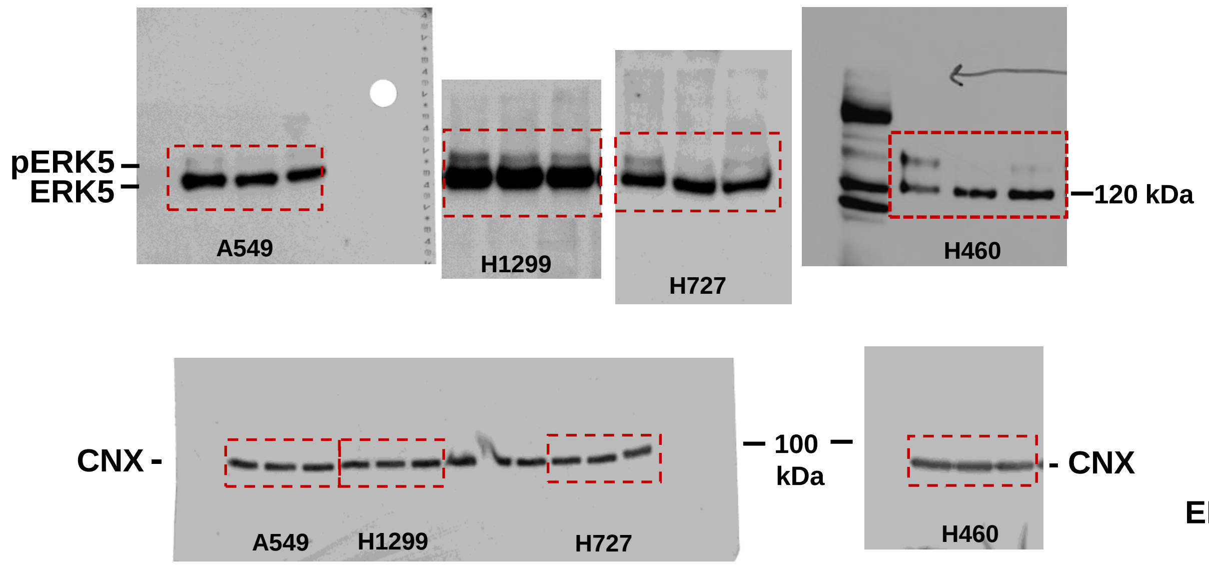


## Supplementary Figure 3d

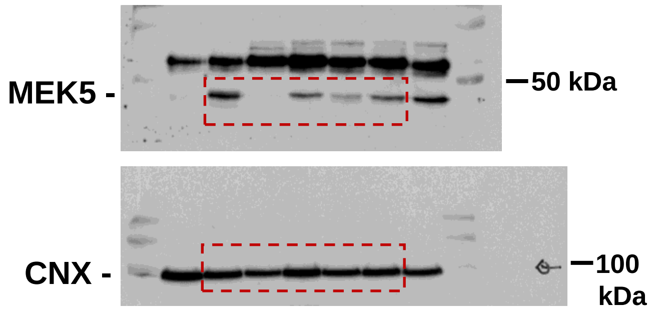


## Supplementary Figure 3e

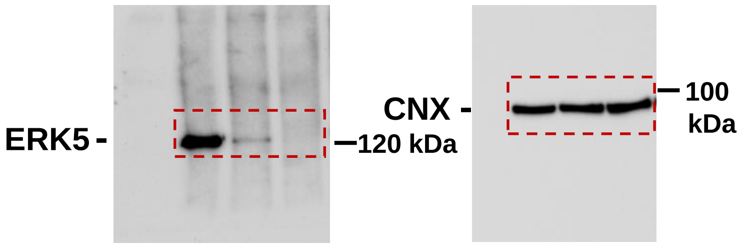

## Supplementary Figure 4a

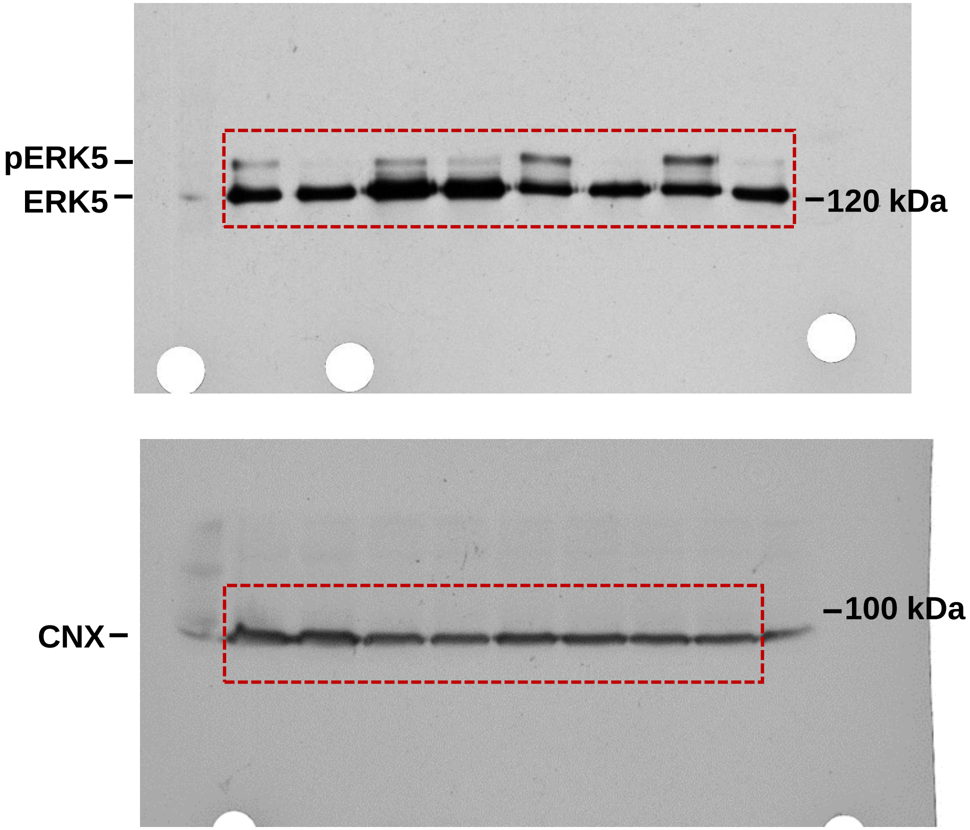


## Supplementary Figure 4c

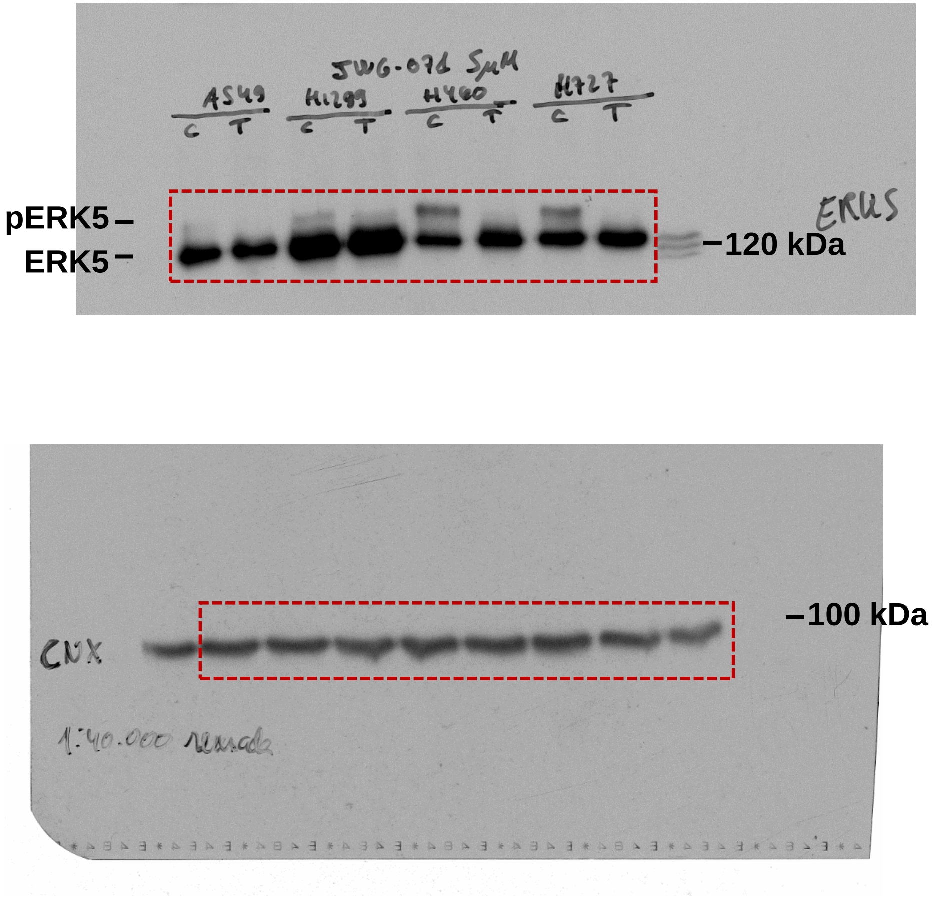

# Supplementary Figure 4e

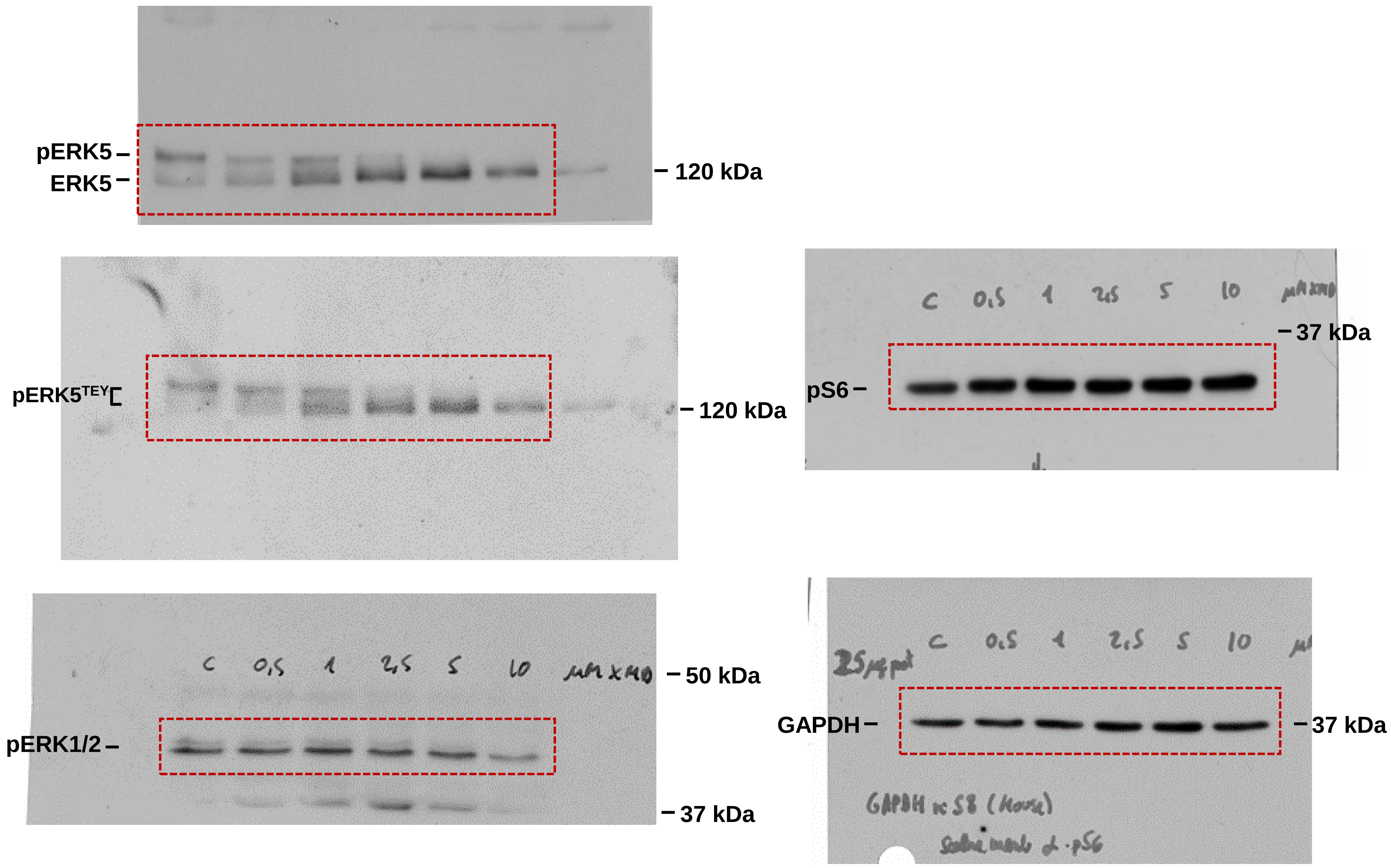

Supplementary Figure 5

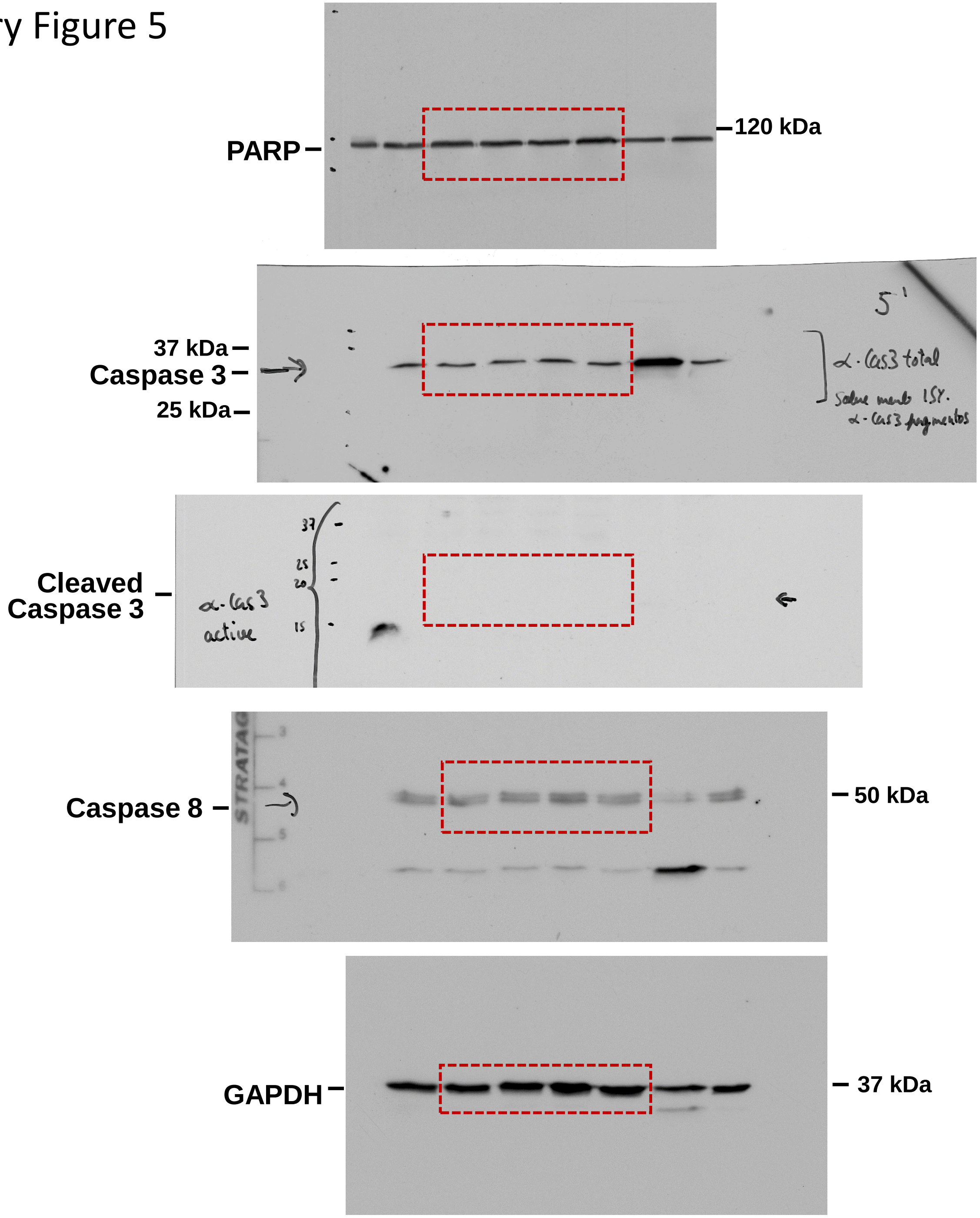

# Supplementary Figure 6

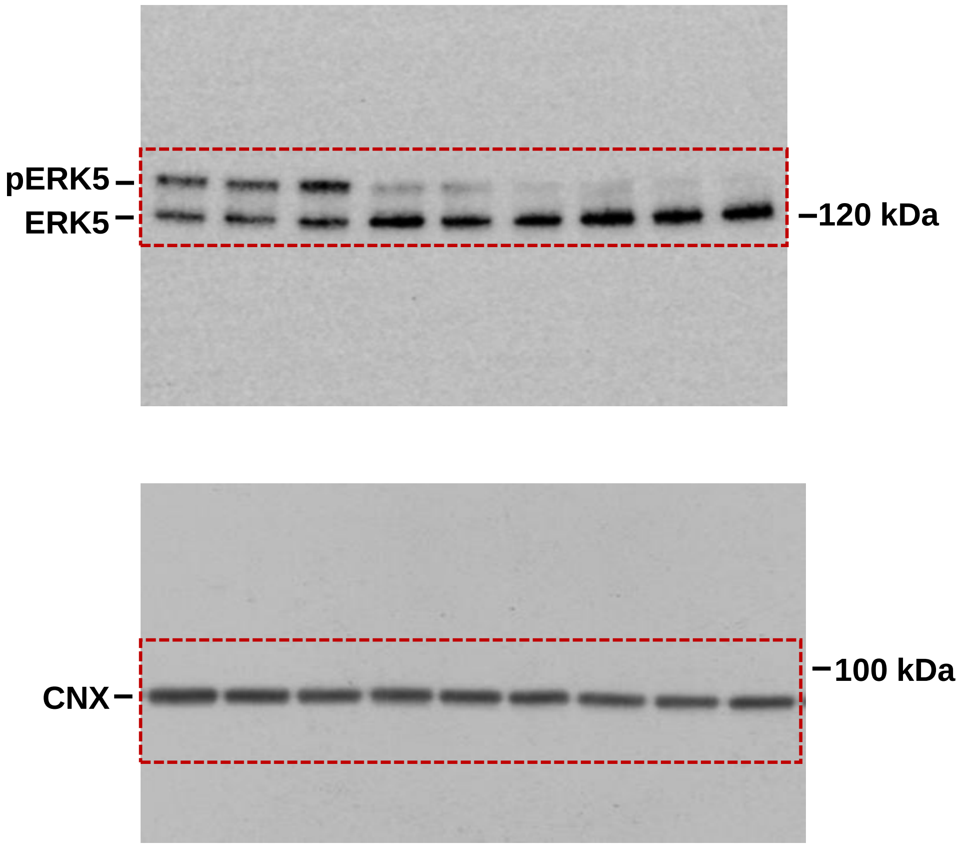

Supplement: Supplementary file 1 — Supplementary Information [file 41698_2021_218_MOESM1_ESM.pdf]
