# Supplementary material for: Clinical, genetic and pharmacological data support targeting the MEK5/ERK5 module in lung cancer
Source: NPJ Precis Oncol. 2021 Aug 17;5:78. doi: 10.1038/s41698-021-00218-8 (PMC8371118; doi:10.1038/s41698-021-00218-8)
Supplement: Supplementary file 2 — Reporting Summary [file 41698_2021_218_MOESM2_ESM.pdf]

nature research

Corresponding author(s): DBPR, NPJPRECISIONONCOLOGY-00738R

Last updated by author(s): Jun 28, 2021

Reporting Summary

Nature Research wishes to improve the reproducibility of the work that we publish. This form provides structure for consistency and transparency in reporting. For further information on Nature Research policies, see our Editorial Policies and the Editorial Policy Checklist.

Statistics

For all statistical analyses, confirm that the following items are present in the figure legend, table legend, main text, or Methods section.

n/a	Confirmed	
☐	☒	The exact sample size (n) for each experimental group/condition, given as a discrete number and unit of measurement
☐	☒	A statement on whether measurements were taken from distinct samples or whether the same sample was measured repeatedly
☐	☒	The statistical test(s) used AND whether they are one- or two-sided<br>*Only common tests should be described solely by name; describe more complex techniques in the Methods section.*
☐	☒	A description of all covariates tested
☐	☒	A description of any assumptions or corrections, such as tests of normality and adjustment for multiple comparisons
☐	☒	A full description of the statistical parameters including central tendency (e.g. means) or other basic estimates (e.g. regression coefficient) AND variation (e.g. standard deviation) or associated estimates of uncertainty (e.g. confidence intervals)
☐	☒	For null hypothesis testing, the test statistic (e.g. F, t, r) with confidence intervals, effect sizes, degrees of freedom and P value noted<br>*Give P values as exact values whenever suitable.*
☒	☐	For Bayesian analysis, information on the choice of priors and Markov chain Monte Carlo settings
☒	☐	For hierarchical and complex designs, identification of the appropriate level for tests and full reporting of outcomes
☒	☐	Estimates of effect sizes (e.g. Cohen's d, Pearson's r), indicating how they were calculated

Our web collection on statistics for biologists contains articles on many of the points above.

Software and code

Policy information about availability of computer code

Data collection	Publicly available data from cBioportal (https://www.cbioportal.org/) and KMplotter (https://kmplot.com/) are used. The studies selected and the parameters used for our analyses are fully described in the methods section of the manuscript and the Code Availability statement.
Data analysis	Publicly available data from cBioportal (https://www.cbioportal.org/) and KMplotter (https://kmplot.com/) are used. The studies selected and the parameters used for our analyses are fully described in the methods section of the manuscript and the Code Availability statement.

For manuscripts utilizing custom algorithms or software that are central to the research but not yet described in published literature, software must be made available to editors and reviewers. We strongly encourage code deposition in a community repository (e.g. GitHub). See the Nature Research guidelines for submitting code & software for further information.

Data

Policy information about availability of data

All manuscripts must include a data availability statement. This statement should provide the following information, where applicable:

- Accession codes, unique identifiers, or web links for publicly available datasets
- A list of figures that have associated raw data
- A description of any restrictions on data availability

Relevant data supporting the findings of this study are available within the paper and its supplementary information files.

Field-specific reporting

Please select the one below that is the best fit for your research. If you are not sure, read the appropriate sections before making your selection.

☒ Life sciences ☐ Behavioural & social sciences ☐ Ecological, evolutionary & environmental sciences

For a reference copy of the document with all sections, see nature.com/documents/nr-reporting-summary-flat.pdf

Life sciences study design

All studies must disclose on these points even when the disclosure is negative.

Sample size	Sample sizes were selected on the basis of previous studies to ensure adequate power of statistical analyses. For animal studies, sample size was chosen according to the minimum number of animals which would give enough statistical power, and according to the Three Rs of Animal Research (Directive 2010/63/EU)
Data exclusions	Animals would be excluded from analysis only if they became ill, or their tumors experienced massive necrosis.
Replication	Experiments were performed at least for duplicate, with success.
Randomization	In the case of testing the effect of the knockdown o knockout of a protein, animals were randomly allocated into experimental groups. In the case of pharmacologycal experiments, animals were divided into experimental groups when the tumor size that they were bearing was around 125 mm3
Blinding	In vitro analyses were not blind but occasionally verified by independent researchers within the group. In vivo selection of animals was random or not (see the comment above)

Reporting for specific materials, systems and methods

We require information from authors about some types of materials, experimental systems and methods used in many studies. Here, indicate whether each material, system or method listed is relevant to your study. If you are not sure if a list item applies to your research, read the appropriate section before selecting a response.

Materials & experimental systems

n/a	Involved in the study	
☐	☒	Antibodies
☐	☒	Eukaryotic cell lines
☒	☐	Palaeontology and archaeology
☐	☒	Animals and other organisms
☒	☐	Human research participants
☒	☐	Clinical data
☒	☐	Dual use research of concern

Methods

n/a	Involved in the study	
☒	☐	ChIP-seq
☐	☒	Flow cytometry
☒	☐	MRI-based neuroimaging

Antibodies

Antibodies used	MEK5: MEK5 polyclonal antibody; cat. nº ADI-KAP-MA003; lot. nº 01031958. Enzo life Sciences (Farmingdale, NY. USA).<br>GAPDH: GAPDH (FL-335) mouse polyclonal; cat. nº sc-25778; lot nº J0212. Santa Cruz Biotechnology (Santa Cruz, CA, USA).<br>PARP: PARP-1 (F-2); cat. nº sc-8007; lot nº A0617. Santa Cruz Biotechnology (Santa Cruz, CA, USA).<br>pCDK1: p-Cdc2 p34 (Tyr 15); sc-7989. Santa Cruz Biotechnology (Santa Cruz, CA, USA).<br>Wee1: Wee1 (H-300); cat. nº sc-9037; lot nº L1905. Santa Cruz Biotechnology (Santa Cruz, CA, USA).<br>Cleaved Caspase-3: Cleaved Caspase-3 (Asp 175) (5A1E) rabbit mAb; cat. nº 9664; lot nº 21. Cell Signaling Technology (Danvers, MA, USA).<br>pERK1/2: Phospho-p44/42 MAPK (ERK1/2) (Thr202/Tyr204) (E10)Mouse mAb; cat. nº 9106; lot nº 43. Cell Signaling Technology (Danvers, MA, USA).<br>pS6: Phospho-S6 Ribosomal Protein (Ser240/244) Antibody; cat. nº 2215. Cell Signaling Technology (Danvers, MA, USA).<br>p27: p27 Kip1 (D69C12) XP® Rabbit mAb; cat. nº 3686; lot nº 5. Cell Signaling Technology (Danvers, MA, USA).<br>Caspase-8: Caspase-8; cat. nº 551242; BD Biosciences (San Jose, CA, USA).<br>Caspase-3: Purified Mouse Anti-Human Caspase-3, Monoclonal (19/Caspase-3/CPP32); cat nº 610323; lot nº 17922. BD Biosciences (San Jose, CA, USA).<br>Cyclin A: Purified Mouse Anti-Human Cyclin A, Clone 25/Cyclin A; cat nº 611268; lot nº 5023928. BD Biosciences (San Jose, CA, USA).<br>BUBR1: Purified Mouse Anti-Human BUBR1, Clone 9/BUBR1; cat nº 612503; BD Biosciences (San Jose, CA, USA).<br>Rb: Purified Mouse Anti-Human Retinoblastoma Protein, Monoclonal (G3-245); cat. nº 554136. Lot nº 36721. BD Biosciences (San Jose, CA, USA).<br>pRb: Purified Mouse anti-Rb (pS807/pS811); cat nº 558389; BD Biosciences (San Jose, CA, USA).<br>Calnexin: Rabbit polyclonal anti-calnexin; cat. nº SPA-860; lot nº B509422. Stressgen Bioreagents (Victoria, BC, Canada).

	pH3: Anti-phospho-Histone H3 (Ser10) Antibody, Mitosis Marker; cat. nº 06-570; lot nº 3076467. EMD Millipore Corp., (USA). Ki-67: Rabbit Anti-Human Ki-67 Monoclonal Antibody (Clone SP6); cat. nº MAD-000310QD. Vitro Master Diagnostica (Granada, Spain).
Validation	antibodies were used as described in the manuscript.

Eukaryotic cell lines

Policy information about cell lines

Cell line source(s)	cell lines were obtained from the ATCC
Authentication	Their authenticity was checked by STR profiling
Mycoplasma contamination	Mycoplasma Testing was periodically made in order to keep cells negative for mycoplasma in the four cell lines used for the study
Commonly misidentified lines (See ICLAC register)	The cell lines used are not misidentified cells.

Animals and other organisms

Policy information about studies involving animals; ARRIVE guidelines recommended for reporting animal research

Laboratory animals	Balb/c nude /J mice (Charles River, Wilmington, MA, USA), females, six weeks old.
Wild animals	The study did not involve wild animals
Field-collected samples	The study did not involve samples collected from the field
Ethics oversight	All animals were manipulated by authorized personal at the animal facility following legal and institutional guidelines. Experimentation was approved by the University of Salamanca Bioethics Committee.

Note that full information on the approval of the study protocol must also be provided in the manuscript.

Flow Cytometry

Plots

Confirm that:

- [x] The axis labels state the marker and fluorochrome used (e.g. CD4-FITC).
- [x] The axis scales are clearly visible. Include numbers along axes only for bottom left plot of group (a 'group' is an analysis of identical markers).
- [x] All plots are contour plots with outliers or pseudocolor plots.
- [x] A numerical value for number of cells or percentage (with statistics) is provided.

Methodology

Sample preparation	This is indicated in the Materials and Methods section of the manuscript.
Instrument	BD Accuri C6 flow cytometer
Software	BD Accury C6 software
Cell population abundance	No specific sorting of cell populations was performed
Gating strategy	Gates were applied to density plots to exclude debris populations or to positively select populations in G0/G1, S or G2/M cell cycle phases for further quantitation.

- [ ] Tick this box to confirm that a figure exemplifying the gating strategy is provided in the Supplementary Information.
